# An Operon of Three Transcriptional Regulators Controls Horizontal Gene Transfer of the Integrative and Conjugative Element ICE*clc* in *Pseudomonas knackmussii* B13

**DOI:** 10.1371/journal.pgen.1004441

**Published:** 2014-06-19

**Authors:** Nicolas Pradervand, Sandra Sulser, François Delavat, Ryo Miyazaki, Iker Lamas, Jan Roelof van der Meer

**Affiliations:** Department of Fundamental Microbiology, University of Lausanne, Lausanne, Switzerland; Universidad de Sevilla, Spain

## Abstract

The integrative and conjugative element ICE*clc* is a mobile genetic element in *Pseudomonas knackmussii* B13, and an experimental model for a widely distributed group of elements in *Proteobacteria*. ICE*clc* is transferred from specialized transfer competent cells, which arise at a frequency of 3-5% in a population at stationary phase. Very little is known about the different factors that control the transfer frequency of this ICE family. Here we report the discovery of a three-gene operon encoded by ICE*clc*, which exerts global control on transfer initiation. The operon consists of three consecutive regulatory genes, encoding a TetR-type repressor MfsR, a MarR-type regulator and a LysR-type activator TciR. We show that MfsR autoregulates expression of the operon, whereas TciR is a global activator of ICE*clc* gene expression, but no clear role was yet found for MarR. Deletion of *mfsR* increases expression of *tciR* and *marR*, causing the proportion of transfer competent cells to reach almost 100% and transfer frequencies to approach 1 per donor. *mfsR* deletion also caused a two orders of magnitude loss in population viability, individual cell growth arrest and loss of ICE*clc*. This indicates that autoregulation is an important feature maintaining ICE transfer but avoiding fitness loss. Bioinformatic analysis showed that the *mfsR-marR-tciR* operon is unique for ICE*clc* and a few highly related ICE, whereas *tciR* orthologues occur more widely in a large variety of suspected ICE among *Proteobacteria*.

## Author Summary

Integrative and conjugative elements (ICEs) are a relatively newly recognized class of mobile elements in bacteria, which integrate at one or more positions in a host chromosome, can be excised, circularized, and transfer by conjugation to a new recipient cell. Genome sequencing indicated that ICEs often carry genes with potential adaptive functions for the host. Various ICE-types have been described and ICE*clc* is a useful model for a wide class of elements found in *Beta*- and *Gammaproteobacteria*. Because ICEs normally remain “silent” in the host chromosome and often lack selectable markers, their lifestyle is difficult to study. One of the characteristics of ICE*clc* is that transfer is initiated in only 3-5% of donor cells in a population during stationary phase. Here, we describe an operon of three regulatory genes, two of which control the transfer initiation of ICE*clc*. Our findings suggest that the low transfer rate results from the repression of an activator and that this is essential to minimize the deleterious effect of hyper-activation of transfer initiation. While the individual regulatory genes are quite common on ICEs, they rarely occur in this configuration.

## Introduction

**Figure 1:**
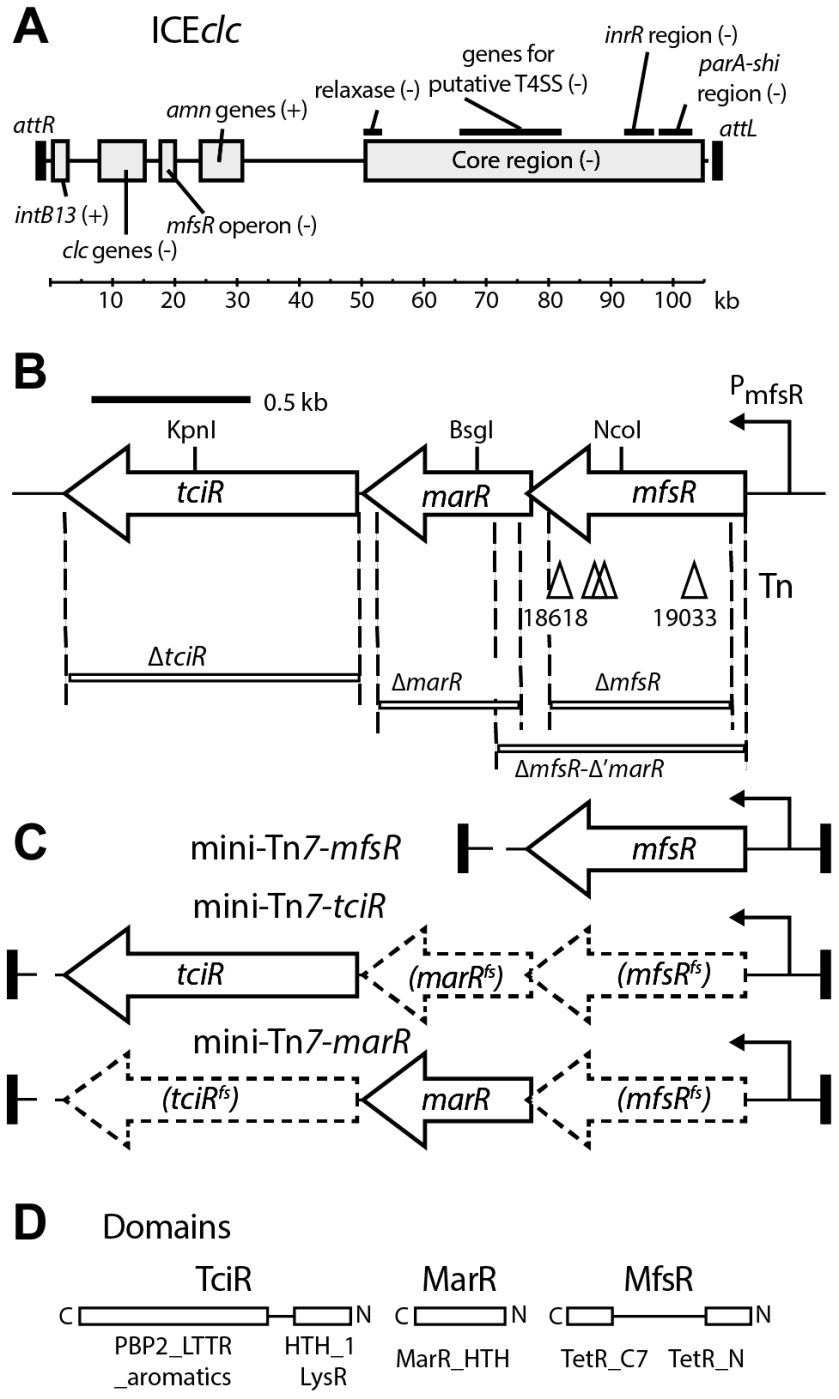
Schematic overview of ICE*clc* and the location of the genes relevant to this study. (A) ICE*clc* integrated form with the two flanking 18-bp repeats (as black rectangles, *attR* and *attL*). Previously determined gene regions are placed at their approximate location. Important functional regions are depicted as grey boxes accompanied by legends. + or -, indicate the orientation of the coding strand for the respective gene(s) (the + strand being the one of *intB13*). *clc* genes, chlorocatechol degradation; *amn* genes, 2-aminophenol degradation. kb, kilo-basepairs. (B) Detail of the *mfsR* operon. Arrows, predicted open reading frames (the right-to-left orientation indicates the minus strand). Triangles, positions of the Tn5-mediated kanamycin gene insertions (nucleotide positions indicated below, according to the AJ617740.2 numbering). Regions deleted in this study are displayed as white bars with the names of the mutations noted below. The hooked arrow indicates repression by the *mfsR* gene product on the P_mfsR_ promoter. (C) Detail of the fragments inserted by mini-Tn*7* delivery for complementation of *mfsR* and *tciR* deletions. (D) BlastP-predicted domains for each of the three regulatory genes in the *mfsR* operon. C and N, carboxy and amino terminus, respectively.

Comparisons between ever-increasing numbers of sequenced genomes reveal the large extent to which prokaryotic genomes have undergone horizontal gene transfer (HGT) [1]-[5]. HGT has traditionally been viewed as the consequence of natural transformation, or of the action of mobile elements such as conjugative plasmids and phages [6], [7]. During the last decade, however, other types of mobile genetic elements such as integrative and conjugative elements (ICEs) have been recognized, which are widespread and thus may significantly contribute to HGT [8]-[13]. In contrast to phages and plasmids, however, we still know little about the life styles of the diverse ICE types, their modes of self-transfer and regulatory pathways controlling self-transfer. Like temperate phages, ICEs mostly exist in an integrated form at one or more specific sites in the host's chromosome (often in genes for tRNA), and are vertically transmitted to daughter cells by chromosome replication and segregation [8], [10], [14]. In order to transfer horizontally, ICEs excise themselves by site-specific recombination (*attL* and *attR*, Figure 1). This produces a circular double-stranded DNA molecule, which can transfer by conjugation to a new recipient cell, where it can reintegrate [14]. Autonomous plasmid-like replication of the excised form may occur [15]-[17], but is not required for the transfer itself.

The regulatory mechanisms that control the switch from integrated to excised state vary widely among different ICE types insofar as this has been studied. In several ICEs, this switch is the consequence of a cascade of a variety of regulatory factors, such as PhrI/RapI and ImmR/ImmA in ICE*Bs1* [18], SetR/SetCD in ICE*SXT* [19], [20], KorSA/Pra in pSAM2 [21]-[23] or QseM/TraR in ICE*MlSym* ^R7^ [24], [25]. Most wild-type ICEs transfer at low frequencies (i.e., less than 1 per 10^3^ donors), suggesting that the regulatory cascades keep extremely tight control and allow only a small subset of cells in a population to follow the path of ICE excision and transfer, but the need for such tight control is *a priori* unclear. This bistability is most pronounced and well-studied for a model ICE named ICE*clc* in *Pseudomonas* [26], [27], which is evolutionary very distinct from the afore-mentioned ICEs [8], [28]. ICE*clc* is originally found in two copies in *Pseudomonas knackmussii* B13 and is member of a family of ICE*clc*-like elements widely distributed among proteobacterial species [29]. ICE*clc* is integrated at the 3′-end of *tRNA^Gly^* genes but can excise itself by the action of the IntB13 integrase encoded on the element (Figure 1A). Expression of *intB13* in the integrated form is under control of the promoter P_int_, which by single cell reporter gene analysis was shown to become active only in 3-5% of a bacterial population during stationary phase [26]. Direct single cell visualization further confirmed that only cells which express reporter gene fused to P_int_ above a threshold are capable of transferring ICE*clc* to new recipients, a bistable state which we recently named “transfer competence” (tc) [30]. Irrespective of the success of ICE*clc* transfer, tc cells can only divide a few times once they re-enter exponential phase before they arrest growth. We recently showed that this is due to the expression of the ICE*clc* genes *shi* and *parA* [30]. Expression of *intB13* is dependent on a variety of factors, most notably a gene named *inrR* (Figure 1A), which itself is also bistably expressed [26]. Both *inrR* and *intB13* expression are dependent on the abundance of the stationary phase sigma factor RpoS, with cells having highest RpoS levels being more likely to activate P_int_ and P_inR_ [29]. RpoS and InrR are important for activating ICE*clc* excision and transfer, but are not sufficient. Therefore, we hypothesized that additional factors are necessary for the tc state to develop [29].

In this study, we report a locus of three consecutive regulatory genes on ICE*clc*, which is essential for controlling its transfer. The locus was uncovered by random transposon mutagenesis, and further studied by creation of deletion mutants and complementation. The effect of mutations was studied at the level of ICE*clc* expression through microarray hybridizations, RT-PCR and reporter gene-based single cell fluorescence microscopy, and further in ICE*clc* transfer assays. Fitness of mutants compared to wild-type was examined in growth assays and individual cell fates were followed by microscopy. Bioinformatics was used to analyze the configuration of the ICE*clc* regulatory locus within this ICE family, and to possibly reconstruct the steps that may have led to selection of the specific regulatory control mechanism of ICE*clc*. The results of our study help to explain why a careful balance has to be maintained between ICE transfer frequency and fitness loss.

## Results

### Discovery of an ICE*clc* transfer control locus by transposon mutagenesis

**Figure 2:**
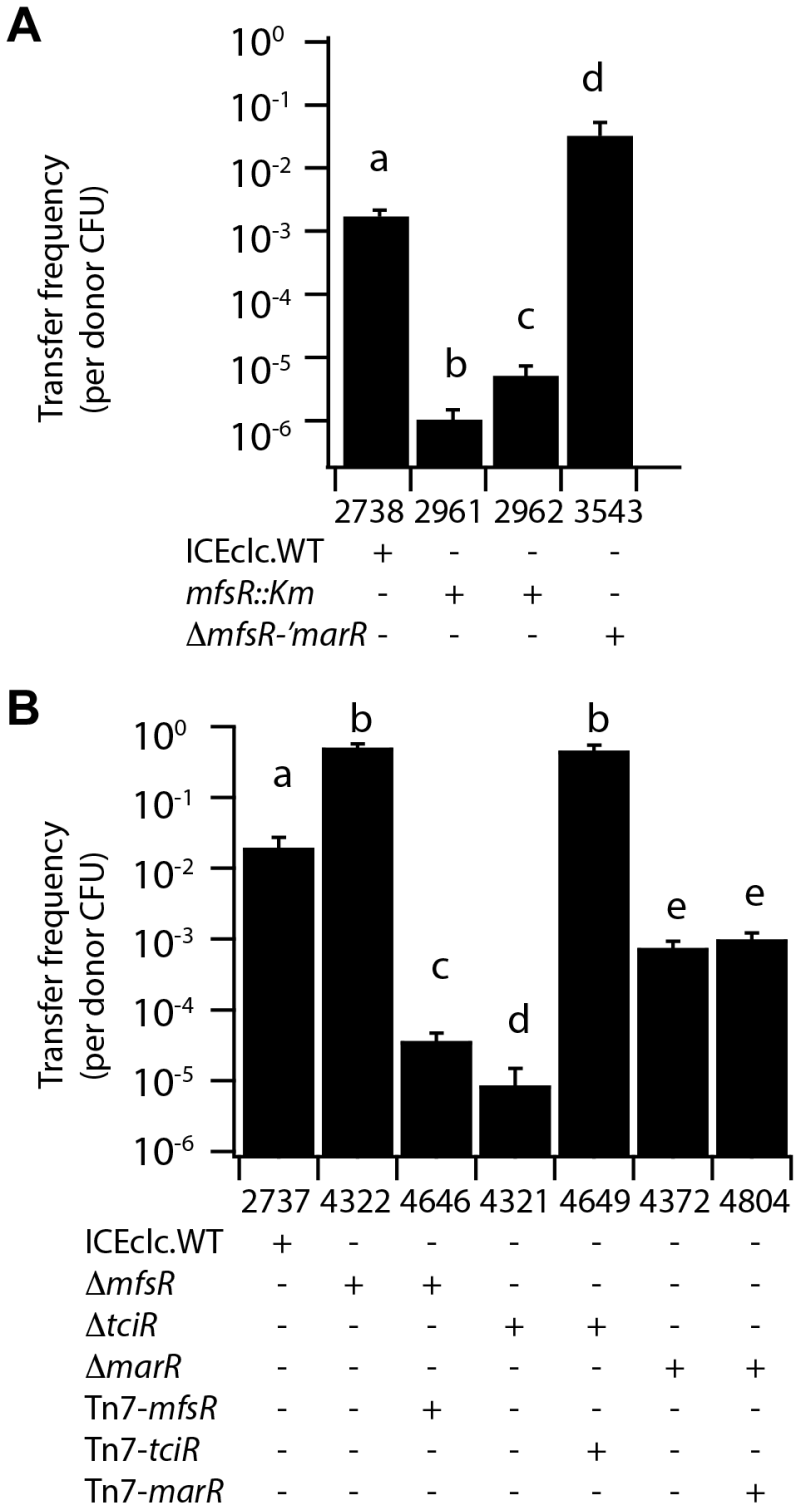
ICE*clc* transfer frequencies from *P. putida* UWC1 donors with different ICE*clc* genotypes. (A) and (B), Independently carried out transfer experiments using the indicated strain sets. Bars show mean transfer frequencies as transconjugant colony forming units (CFU/ml, growing on 3CBA, Km- or Gm-resistant) per donor CFU/ml from biological triplicates, and the corresponding standard deviations. Letters above bars indicate statistically significantly different groups per panel in an Anova with post hoc Tukey-Kramer test (P<0.001), with the same letter pointing to the absence of statistically significant differences.

In order to discover ICE*clc*-located factors involved in its self-transfer, a library of *P. knackmussii* B13 mutants was generated by using random Tn*5* mutagenesis [31]. Next, we recovered ICE*clc* elements with Km-insertions by conjugating the pool of B13 mutants *en masse* to *Pseudomonas putida* UWC1 and selecting for Km-resistant *P. putida* (Figure S1). We hypothesized that mutant ICE*clc* with insertions in genes implicated in self-transfer could still be transferred to UWC1, when the second copy of ICE*clc* in the same B13 donor cell is intact and complements transfer of the mutant copy. A total of 1920 Km-resistant *P. putida* transconjugants was recovered and subsequently conjugated each individually with a second *P. putida* recipient, resistant to nalidixic acid (Figure S1). For those conjugations in which no Km- and nalidixic acid-resistant transconjugant growth was detected, the corresponding *P. putida* donor was recovered and the location of the Km^R^-gene insertion on ICE*clc* was mapped. A total of 18 clones was recovered, which had insertions in an ICE*clc* open reading frame numbered *orf18502*, that we renamed *mfsR* (Figure 1B). Surprisingly, apart from one donor with an insertion in *intB13*, no other mutants with impaired ICE*clc* transfer were found in this screening. The Km^R^-gene had been inserted in four different positions in *mfsR*, at ICE*clc* nucleotide positions 19033, 18758, 18730 and 18618 (Figure 1B, accession number: AJ617440.2). This suggests that transposon insertions in strain B13 were sufficiently frequent to cover all genes, but that the selection procedure was biased for the recovery of the *mfsR* insertion, which may have been due to the function of *mfsR* as regulator in ICE*clc* transfer (see below). Alternatively, it is possible that insertions in ICE*clc* genes needed for transfer might not be efficiently complemented by the second ICE*clc* copy and would thus be underrepresented in the *P. putida* library. Frequencies of ICE*clc* transfer of the strains *P. putida* UWC1-2961 (Km^R^-gene insertion at 19033) and UWC1-2962 (insertion at 18618) in a filter-based conjugation assay were 10^3^-fold and 10^2^-fold lower than of a *P. putida* with one integrated wild-type ICE*clc* copy, respectively (Figure 2A).

### *mfsR* is part of an operon formed by three consecutive transcriptional regulators

Closer inspection indicated *mfsR* to be the first open reading frame in a series of three consecutive transcriptional regulators, previously designated as *orf18502, orf17984* and *orf17162* (Figure 1B). *mfsR* encodes a TetR-like regulator harboring helix-turn-helix motifs TetR_N and TetR_C_7 (pfam0040 and pfam14246, respectively, see Figure 1D). The *orf17984* gene overlaps with the end of the *mfsR* open reading frame by 4 bp and encodes a putative regulator of the MarR family (smart00347 HTH_MARR motif). The last gene of this cluster starts 24 bp downstream of the stop codon of *orf17984* and is predicted to code for a LysR-type transcriptional regulator, harboring an N-terminal HTH_1 motif (pfam00126) and a C-terminal substrate-binding domain (PBP2_LTTR_aromatics_like; cd08414). The gene *orf17162* was renamed *tciR* (transfer competence inducer regulator) in anticipation of the results described further below. Reverse transcription of *P. putida* UWC1 (ICE*clc*) RNA isolated from exponential phase-grown cells, followed by specific PCR amplification confirmed that the three genes are transcribed on the same mRNA, which ends downstream of *tciR* (Figure S2). This implies that *mfsR-marR-tciR* form a single polycistronic unit.

### Effects of *tciR, marR*, and *mfsR* deletions on ICE*clc* transfer

In order to more precisely investigate the role of the three regulators on ICE*clc* transfer, their open reading frames were each individually and partially deleted in separate strains, namely *P. putida* UWC1 (ICE*clc*-Δ*mfsR*, strain 4322), UWC1 (ICE*clc*-Δ*marR*, strain 4372), UWC1 (ICE*clc*-Δ*mfsR*-Δ'*marR*, strain 3453) and *P. putida* UWC1 (ICE*clc*-Δ*tciR*, strain 4321) (Figure 1B, Table 1). ICE*clc* transfer frequencies in plate-mating assays with a gentamicin-resistant *P. putida* UWC1 as recipient were 2·10^3^-fold lower for UWC1 donors with ICE*clc* having an internal deletion in *tciR* compared to intact ICE*clc* (Figure 2B). Complementation of the ICE*clc*-Δ*tciR* mutation with a single copy mini-Tn*7* transposed fragment containing the *tciR* gene under the P_mfsR_-promoter (strain 4649, Figure 1C) restored transfer, even to much higher levels than wild-type ICE*clc* (Figure 2B).

**Table 1:** Strains used in this study and their specifications.

Strain name	Strain collection number	Relevant characteristics	Reference or source
*Escherichia coli* DH5α			[36]
*E. coli* DH5α λpir			V. de Lorenzo
*E. coli* BW20767/pRL27	1853	*tra* ^+^, pRL27 containing hyperactive mini-Tn^5^ element (*oriV*, Km^R^).	[31]
*Pseudomonas knackmussii* B13	78	Original host of ICE*clc* (2 identical copies).	[43]
*Pseudomonas putida* UWC1	1291	plasmid-free derivative of *P. putida* KT2440, Rif^R^	[44]
*P. putida* UWC1 (Nal)		Spontaneous Nal^R^-mutant of 1291.	This study
*P. putida* UWCGC	2744	Single copy mini-Tn*7*-P_tac_-*echerry* insertion, Gm^R^	[33]
*P. putida* UWC1	2756	Single copy mini-Tn*5*-jimX-*gfp* insertion, Km^R^	[45]
*P. putida* UWC1 (ICE*clc*)	2737	Derivative of strain 1291 with one ICE*clc* copy integrated into *tRNA^gly^*-5.	[45]
*P. putida* UWC1 (ICE*clc*)	2738	As 2737, but integrated into *tRNA^gly^*-6.	[45]
*P. putida* UWC1 (ICE*clc*-Km^R^_19033_)	2961	Transposon mutant of strain 2737 with a Km^R^-gene inserted at nucleotide position 19033 in ICE*clc*.	This study
*P. putida* UWC1 (ICE*clc*-Km^R^_18618_)	2962	Transposon mutant of strain 2737 with a Km^R^ -gene inserted at nucleotide position 18618 in ICE*clc*.	This study
*P. putida* UWC1 (ICE*clc*-Δ*mfsR*-Δ*'marR*)	3453	Derivative of strain 2737 with *mfsR* and part of *marR* deleted (from nucleotide position 18395 to 19166).	This study
*P. putida* UWC1 (ICE*clc*) + P_int_-*gfp*/P_inR_-*echerry*	3531, 3532, 3533	Derivatives of strain 2737 with single copy random insertion of a mini-Tn-P_int_-*gfp*/P_inR_-*echerry*, Km^R^	This study
*P. putida* UWC1 (ICE*clc*-Δ*tciR*)	4321	Derivative of strain 2737 with an internal deletion in *tciR* (from nucleotide position 17164 to 17985).	This study
*P. putida* UWC1 (ICE*clc*-Δ*mfsR*)	4322	Derivative of strain 2737 with an internal deletion in *mfsR* (from nucleotide position 18581 to 19143).	This study
*P. putida* UWC1 (ICE*clc*-Δ'*marR*)	4372	Derivative of strain 2737 with an internal deletion in *marR* (from nucleotide position 18032 to 18468).	This study
*P. putida* UWC1 (ICE*clc*-Δ*mfsR*) + P_int_-*gfp*/P_inR_-*echerry*	4469, 4470, 4471	Derivatives of strain 4322 with single copy random insertion of a mini-Tn-P_int_-*gfp*/P_inR_-*echerry*, Km^R^	This study
*P. putida* UWC1 (ICE*clc*-Δ*marR*) + P_int_-*gfp*/P_inR_-*echerry*	4475, 4476, 4477	Derivatives of strain 4372 with single copy random insertion of a mini-Tn-P_int_-*gfp*/P_inR_-*echerry*, Km^R^	This study
*P. putida* UWC1 (ICE*clc*-Δ*tciR*) + P_int_-*gfp*/P_inR_-*echerry*	4479, 4480, 4481	Derivatives of strain 4321 with single copy random insertion of a mini-Tn-P_int_-*gfp*/P_inR_-*echerry*, Km^R^	This study
*P. putida* UWC1 (ICE*clc*-Δ*mfsR*, *gfp*)	4612	Derivative of 4322 having a *gfp* gene inserted downstream of *intB13*.	This study
*P. putida* UWC1 (ICE*clc*-Δ*mfsR*, mini-Tn*7*:*mfsR*)	4646	Derivative of 4322 carrying a single copy mini-Tn*7* insertion of the *mfsR* gene under its own promoter.	This study
*P. putida* UWC1 (ICE*clc*-Δ*tciR*, mini-Tn*7*:*mfsR^fs^-marR^fs^-tciR*)	4649	Derivative of 4321 carrying a single copy mini-Tn*7* insertion of the (frameshifted) *mfsR* and *marR* genes, plus the intact *tciR* gene under the *mfsR* promoter.	This study
*P. putida* UWC1 (ICE*clc*-Δ*marR*, mini-Tn*7*:*mfsR^fs^-marR-tciR^fs^*)	4804	Derivative of 4372 carrying a single copy mini-Tn*7* insertion of the (frameshifted) *mfsR* and *tciR* genes, plus the intact *marR* gene under the *mfsR* promoter.	This study
*P. putida* UWC1 mini-Tn*5*-P_mfsR_-*mcherry*	3482	Single copy insertion of a mini-Tn*5 mfsR* promoter-*mcherry* fusion, Km^R^	This study
*P. putida* UWC1 mini-Tn*7*-*mfsR,* mini-Tn*5*-P_mfsR_-*mcherry*	4302	Derivative of strain 3482 but with a mini-Tn*7* insertion containing the intact *mfsR* gene expressed from its own promoter, Km^R^, Gm^R^	This study
*P. putida* UWC1 (ICE*clc*), mini-Tn*5*-P_mfsR_-*mcherry*	3497	Derivative of 2737, single copy insertion of a mini-Tn*5 mfsR* promoter-*mcherry* fusion, Km^R^	This study
*P. putida* UWC1 (ICE*clc*-Δ*mfsR*), mini-Tn*5*-P_mfsR_-*mcherry*	3606	Derivative of 3453, single copy insertion of a mini-Tn*5 mfsR* promoter-*mcherry* fusion, Km^R^	This study
*P. putida* UWC1 (ICE*clc*-Δ*mfsR*), mini-Tn*7*-*mfsR*, mini-Tn*5*-P_mfsR_-*mcherry*	4282	Derivative of 3606, but with a mini-Tn*7* insertion containing the intact *mfsR* gene expressed from its own promoter, Km^R^, Gm^R^	This study

ICE*clc* transfer frequencies were 27-fold lower for UWC1 donors with ICE*clc* having an internal deletion in *marR* compared to intact ICE*clc* (Figure 2B). Complementation of the ICE*clc*-Δ*marR* mutation with a similar single copy mini-Tn*7*-*marR* insertion did not change transfer rates (strain 4804, Figure 2B). This suggests that the effect of the *marR* deletion on ICE*clc* transfer is rather due to polar disturbance of the expression of the downstream-located *tciR*.

In contrast, ICE*clc* elements with *mfsR* deletions [i.e., *P. putida* UWC1 (ICE*clc*-Δ*mfsR*) and UWC1 (ICE*clc-*Δ*mfsR*-Δ'*marR*)] transferred with 25- and 15-fold higher frequencies than wild type ICE*clc*, respectively (Figure 2A, B). Complementation of the ICE*clc*-Δ*mfsR* mutation with a single copy mini-transposed *mfsR* gene under control of its own promoter reduced ICE*clc*-Δ*mfsR* transfer frequencies by 10^4^-fold, also here much stronger than predicted from wild-type ICE*clc* itself (Figure 2B). These results suggested that *tciR* is the actual regulator of ICE*clc* transfer, and further that *mfsR* is regulating expression of the *mfsR-marR-tciR* operon. Since MfsR is expected to be a repressor, its deletion would lead to higher expression of the downstream genes *marR* and *tciR*, which results in increased ICE*clc* transfer. The effect of the transposon insertions in *mfsR* (i.e., lower ICE*clc* transfer rates, Figure 2A) seems therefore due to a polar effect on *marR-tciR* expression.

### *tciR* encodes a global activator of the genes in the ICE*clc* core region

**Figure 3:**
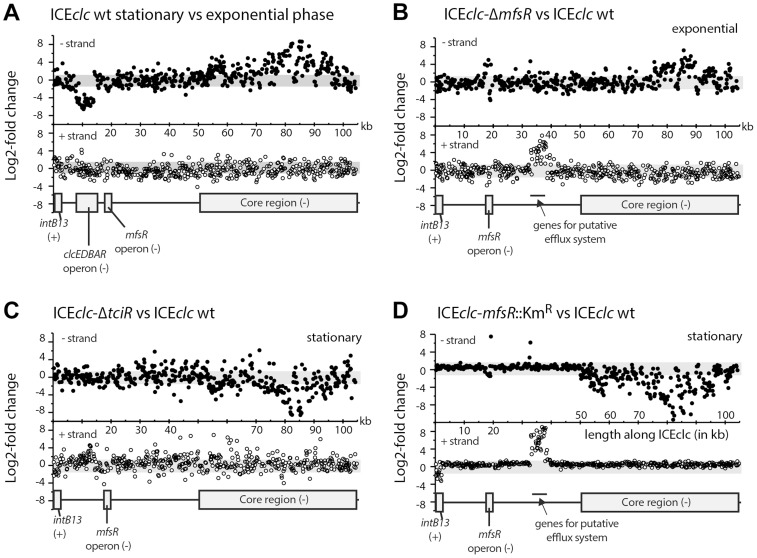
Differential expression of the ICE*clc* gene region from micro-array data in selected mutant ICE*clc* versus wild type in *P. putida* UWC1. (A) Differential expression of the ICE*clc* region between stationary and exponential phase cells of wild type *P. putida* UWC1 (ICE*clc*). (B) Differential expression of the ICE*clc* region between the *mfsR* deletion mutant and wild-type, in exponentially growing cells. (C) Comparison of the *tciR* deletion mutant and wild-type, in stationary phase cells. (D) Comparison of the *mfsR* transposon insertion mutant versus wild-type, in stationary phase cells. Dots indicate the ^2^log-fold change of hybridization signal per microarray probe in the comparison, plotted at their distance along the ICE*clc* sequence (X-axis; in kb). Regions of interest on ICE*clc* are redrawn as grey boxes at the bottom of each section (+ or - indicate the DNA strand on which the region is encoded). Separate displays indicate expression differences on the plus- (open symbols) or the minus-strand (closed symbols). Grey bars in the background indicate the two-fold cut-off level. For a complete set of microarray results, see Figures S3 and S4.

**Figure 4:**
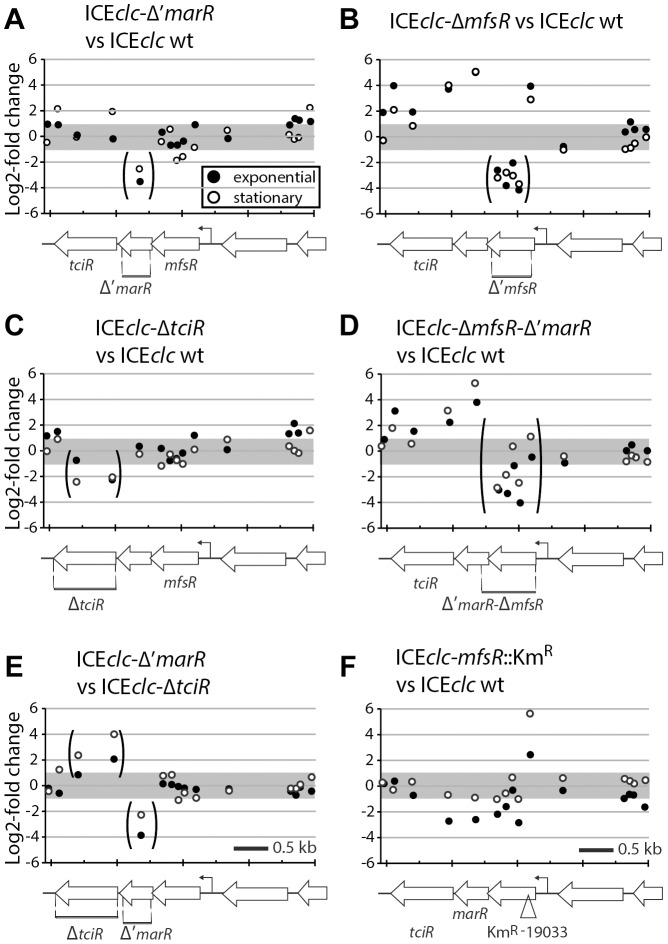
Detailed view on the differential expression of the *mfsR* operon in *P. putida* ICE*clc* wild-type or mutants. (A) *MarR* deletion mutant versus wild-type. (B) *mfsR* deletion mutant versus wild type. (C) *tciR* deletion mutant versus wild-type. (D) *mfsR*-'*marR* deletion mutant versus wild-type. (E) *marR* versus *tciR* deletions. (F) *mfsR-*transposon insertion mutant versus wild-type. Panels show ^2^log-fold change of expression level per microarray probe in this region of ICE*clc* for exponential (dark dots) and stationary phase cells (white dots). Genetic map of the region drawn at the bottom of each section for clarity. Arrows represent genes, deleted regions are indicated by stippled bars and corresponding probes are within brackets.

Next, we examined the effect of regulatory gene deletions on gene expression of ICE*clc* as a whole, using semi-tiling microarray analyses (Figure 3). When *P. putida* UWC1 (ICE*clc*) wild-type cells are growing exponentially on 3-chlorobenzoate (3CBA), expression from the genes in the ICE*clc* core region (roughly the second half of ICE*clc*) plus the integrase *intB13* is silent, whereas they are highly transcribed when cells are in stationary phase (Figure 3A). Among others, the core region encodes genes implicated in ICE*clc* conjugative transfer [32], [33]. *P. putida* with mutant ICE*clc* lacking either *marR* or *tciR* strongly diminished expression in the core region and of the integrase gene in stationary phase when compared to wild type (Figure 3C, Figure S3). Lower core and integrase gene expression explains the lower ICE*clc* transfer rates from these mutants (Figure 2B). In contrast, *mfsR* deletion resulted in much higher expression from the ICE*clc* core genes in exponentially growing cells (Figure 3B), and even slightly higher expression in stationary phase than in wild-type ICE*clc* (Figure S4), which explains the 10- to 100-fold ICE*clc* higher transfer rates (Figure 2B). Expression of the *mfsR-marR-tciR* cluster itself was the same in the *tciR* and *marR* deletion mutants, and no different to the wild-type (Figure 4A, C, E). In contrast, expression of the *mfsR-marR-tciR* cluster was higher in the *mfsR* deletion mutants than in wild-type, both in exponential and stationary phase cells (Figure 4B, D). Since gene expression from ICE*clc* is similar in mutants lacking *mfsR* alone or *mfsR* plus the first 117 bp of *marR* (Figure S4), we conclude that it is the LysR-type regulator encoded by *tciR,* which is the main activator for ICE*clc* core gene expression.

Microarray analysis also helped to understand the behaviour of the *mfsR* Km-insertion mutant (Figure 3D). As for the *tciR* deletion mutant, expression of the ICE*clc* core region and of the integrase was dramatically lower than wild-type in stationary phase cells (Figure 3D, Figure S4). On the other hand, both *mfsR* deletion and *mfsR* Km-insertion mutants showed increased expression of a group of genes on ICE*clc* coding for a putative efflux system (Figure 3B, D). Detailed inspection of *mfsR-marR-tciR* expression in the Km-insertion mutant revealed that the first 160 bp of *mfsR*, upstream of the Km^R^-gene insertion were higher expressed than in wild-type cells (Figure 4F). In contrast, the downstream genes *marR* and *tciR* were lower expressed compared to wild-type and to the *mfsR* deletion mutant (Figure 4B, D, F). This confirmed, therefore, that insertion of the Km^R^-gene had caused a polar effect on expression of *marR* and *tciR*, which explains the strongly diminished expression of the ICE*clc* core genes in stationary phase in the *mfsR* Km-insertion mutant, and decreased ICE*clc* transfer.

**Figure 5:**
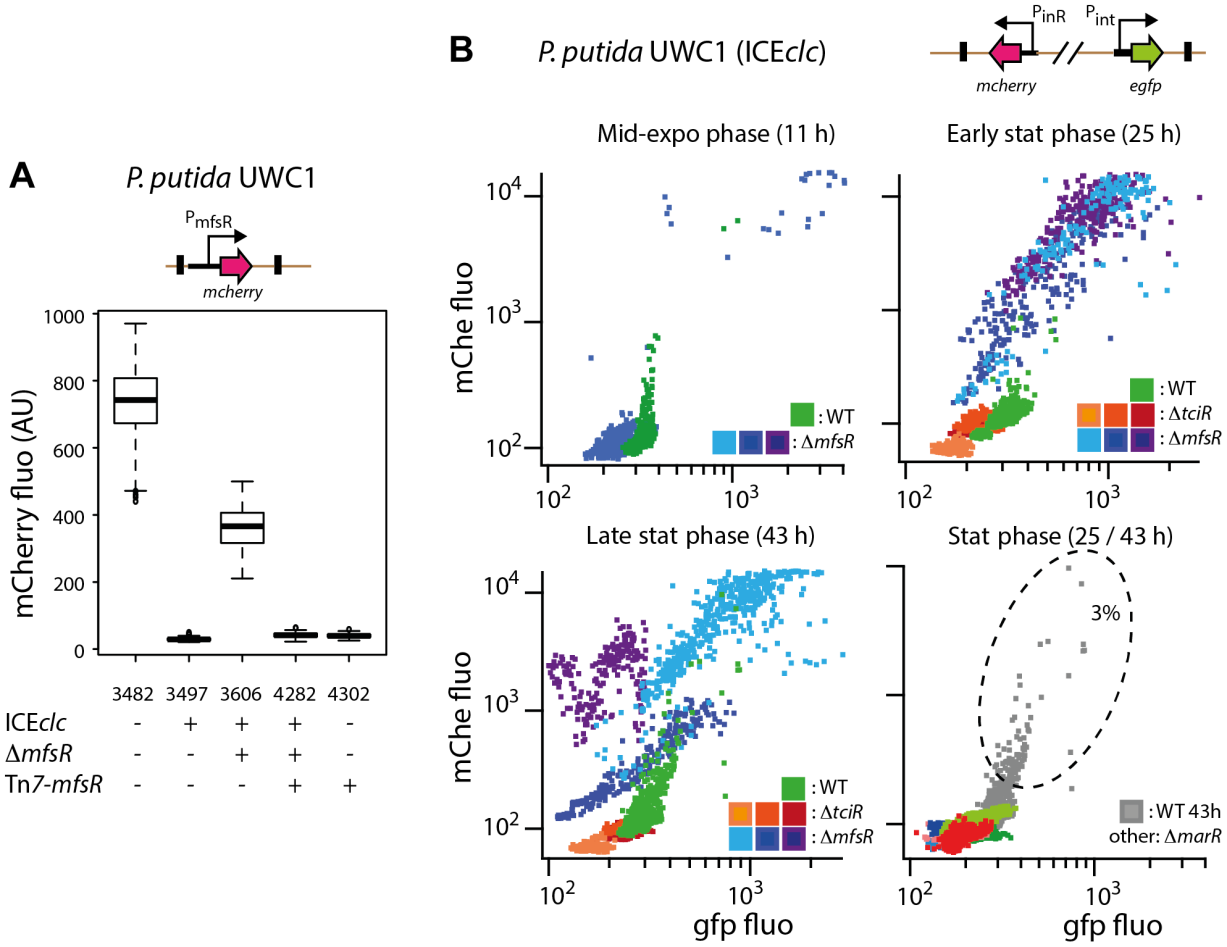
Effect of mutations in the *mfsR* region on the expression of the P_mfsR_-, P_int_- and P_inR_-promoters of ICE*clc* in *P. putida* UWC1. (A) mCherry expression from the *mfsR* promoter added in single copy to the chromosome of the indicated *P. putida* UWC1 strains (relevant genotypes and strain numbers specified below the graph). mCherry expression measured on individual cells (n = 1000) by epifluorescence microscopy in late exponential phase of cultures grown on 10 mM succinate and expressed as box plots (AU, arbitrary units at 20 ms exposure time). (B) Scatter plot of GFP and mcherry fluorescence in single cells of *P. putida* UWC1 (ICE*clc*) wild-type, Δ*mfsR*, Δ*tciR* or Δ*marR* deletions, equipped with a single copy mini-transposon containing the P_int_-*egfp* and P_inR_-*mcherry* fusions. Panels show expression of both markers at different growth phases, as indicated, with colors representing genotypes with independent mini-Tn*5* insertions. Note as example the subpopulation of wild-type cells (dotted ellips) expressing both reporters, compared to the majority of cells in the *mfsR* deletion mutant but a complete absence of such subpopulation in the *tciR* and *marR* deletion mutants.

Inserting the presumed *mfsR* promoter region upstream of a promoterless *mcherry* gene in single copy on the chromosome of *P. putida* UWC1 without ICE*clc* produced strong and homogenous mCherry expression among all cells (Figure 5A, strain 3482). Inserting into this strain a single copy *mfsR* gene expressed from its own promoter abolished mCherry expression (Figure 5A, strain 4302). Expression of mCherry from P_mfsR_ in *P. putida* UWC1 (ICE*clc*) was very low, whereas disruption of *mfsR* on ICE*clc* again resulted in high mCherry expression (Figure 5A, strain 3606). Complementation of this strain by a single copy *mfsR* gene under its own promoter caused repression of mCherry expression (Figure 5A). All these data are consistent with the hypothesis that MfsR is repressing expression of itself and the downstream located *marR* and *tciR* genes.

### *mfsR* deletion leads to an increase in the number of cells activating ICE*clc*

We then tested whether changed ICE*clc* transfer rates and core gene expression were in fact due to changes in the proportion of cells activating ICE*clc*. Hereto, a double promoter-reporter construct, carrying P_int_-*gfp* and P_inR_-*echerry* was inserted in single copy on the chromosomes of *P. putida* UWC1 (ICE*clc*), *P. putida* UWC1 (ICE*clc*-Δ*mfsR*), *P. putida* UWC1 (ICE*clc*-Δ'*marR*) and *P. putida* UWC1 (ICE*clc*-Δ*tciR*) (Table 1). P_int_ and P_inR_ are the respective promoters for the integrase gene *intB13* (integrated form) and the integrase activator gene *inrR*. Previous studies showed that both promoters are active only in a small subpopulation of cells, which are representative for transfer competent cells [26], [29], [30]. Consistent with previous data, the subpopulation of *P. putida* UWC1 (ICE*clc*) wild-type cells expressing P_int_ and P_inR_ in stationary phase suspended cultures represented a few percent (Figure 5B). In contrast, deletion in *mfsR* resulted in 80-100% of cells expressing P_int_- and P_inR_-promoters (Figure 5B). Expression of both promoters in *P. putida* UWC1 (ICE*clc*-Δ*mfsR*) occurred in early stationary phase whereas in wild-type cells their expression is maximal in late stationary phase (Figure 5B). Conversely, *P. putida* UWC1 (ICE*clc*-Δ*tciR*) and *P. putida* UWC1 (ICE*clc*-Δ*marR*) did not produce any detectable P_int_- or P_inR_-expressing cells, neither in exponential nor in stationary phase (Figure 5B). Considering a detection limit by microscopy of ∼1 fluorescent cell among 1000-10,000 non fluorescent cells, the absence of detectable P_int_- or P_inR_-expressing cells in those mutants would be in accordance with absence of ICE*clc* core gene activation on microarrays (Figure 3D) and lower transfer frequencies (Figure 2B).

### Mutants with *mfsR* deletion in ICE*clc* face a strong fitness cost

**Figure 6:**
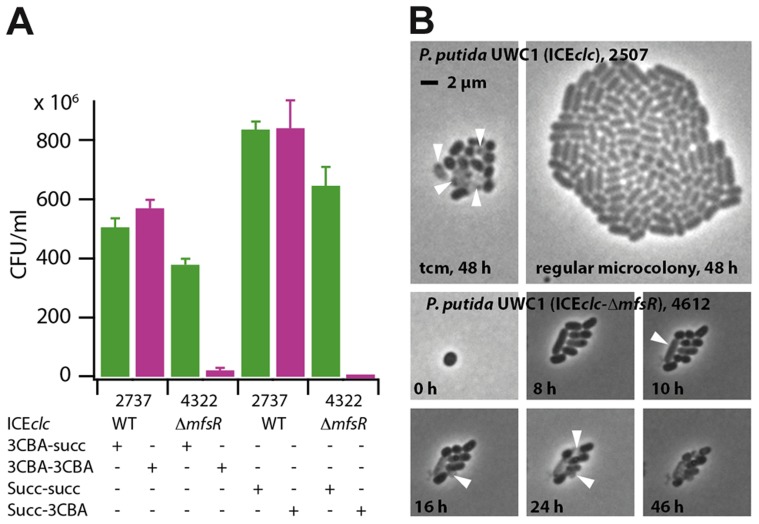
Fitness loss of *P. putida* UWC1 (ICE*clc*) caused by the *mfsR* deletion. (A) Survival of *P. putida* UWC1 (ICE*clc*, 2737) and *P. putida* UWC1 (ICE*clc*-Δ*mfsR*, 4322) pregrown in suspended culture to stationary phase on 3-chlorobenzoate (3CBA) or succinate (succ), and plated from there on 3CBA or succinate agar. (E.g., 3CBA-3CBA, suspended culture on 3CBA, plated on 3CBA agar). Survival expressed as colony forming units (CFU) on the agar plate per ml of stationary phase culture. Data bars indicate the average from independent biological triplicates. Error bars indicate the calculated standard deviation from the average. (B) Phase-contrast micrographs at 1000-fold magnification of microcolonies of *P. putida* UWC1 (ICE*clc*) and *P. putida* (ICE*clc*-Δ*mfsR* P_int_-*egfp*, 4612) growing on agarose surface supplemented with 0.1 mM 3CBA. Shown are a regular stationary phase microcolony of *P. putida* with wild-type ICE*clc* and a transfer competent microcolony (tcm), occurring at 1-3% frequency as reported previously [30]. For comparison, massive lysis (white arrows) and cellular malformations formed in many microcolonies of *P. putida* UWC1 with the *mfsR* deletion.

Given that *P. putida* UWC1 carrying ICE*clc-*Δ*mfsR* transferred at a much higher rate than wild-type ICE*clc*, and also expressed both P_int_- and P_inR_- promoters in almost all cells, we wondered why such mutants did not become selected spontaneously. Both *P. putida* UWC1 (ICE*clc*) wild-type and (ICE*clc-*Δ*mfsR*) displayed statistically indistinguishable generation times during exponential growth on minimal medium with either 3CBA or succinate as carbon source (Table 2), although *P. putida* UWC1 (ICE*clc-*Δ*mfsR*) went through a longer lag phase (Figure S5A). In contrast, the proportion of colony forming units (CFU) in samples taken from stationary phase cultures on 3CBA or succinate and plated on 3CBA solid medium was dramatically reduced for *P. putida* UWC1 (ICE*clc-*Δ*mfsR*) (Figure 6). Whereas the number of colonies formed on 3CBA plates was the same as that on succinate for *P. putida* UWC1 (ICE*clc*), only 0.3-5.6% appeared on 3CBA plates for UWC1 (ICE*clc-*Δ*mfsR*) (Table 2). Also in absolute terms, the number of CFU/ml for UWC1 (ICE*clc-*Δ*mfsR*) cells taken from stationary phase cultures both on 3CBA and succinate was lower than that for UWC1 (ICE*clc*) (Figure 6). Moreover, 8 of 10 tested colonies of UWC1 (ICE*clc-*Δ*mfsR*) grown on MM plates with succinate did no longer amplify the *clcA* gene of ICE*clc* (not shown), the remaining two still being able to grow on 3CBA. Furthermore, half or more of microcolonies formed from UWC1 (ICE*clc-*Δ*mfsR*) with a single copy P_int_-*egfp* insertion showed incidence of malformations and cell lysis, similar to what was reported previously for nutrient-reactivated tc cells [30] (Figure 6B), but cells in the other microcolonies divided with generation times even slightly faster (1.49±0.15 h) than those in microcolonies of *P. putida* UWC1 (ICE*clc*, 1.79±0.09 h). This indicates that the *mfsR* deletion in ICE*clc* imposes a strong fitness cost on *P. putida* UWC1. Survival of UWC1 was restored to wild-type level when the ICE*clc-*Δ*mfsR* was complemented by the mini-Tn*7* inserted *mfsR* gene (Figure S6). In contrast, neither *P. putida* UWC1 (ICE*clc*) with *marR* or *tciR* deletion, nor the mini-Tn*7* complemented strains of *P. putida* UWC1 (ICE*clc*-Δ*tciR*) and (ICE*clc*-Δ*marR*) were impaired in survival compared to UWC1 (ICE*clc*) (Figure S6).

**Table 2:** Effects of the *mfsR* deletion on the growth characteristics of *P. putida* UWC1 carrying ICE*clc*.

Strain		Generation time (min)^1^		Survival rate (%)^2^	
Name	Number	MM Succ	MM 3CBA	MM Succ	MM 3CBA
*P. putida* UWC1 (ICE*clc*)	2737	74.4±6.8^3^	186±11	100±12	113±8.5
*P. putida* UWC1 (ICE*clc*-Δ*mfsR*)	4322	68.2±8.1	184±3	0.3±0.3	5.6±2.5
		p = 0.38^4^	p = 0.77	p = 0.00012	p = 2.96·10^-5^

1) Generation time was calculated as ln2/μ, whereby µ (min^-1^) is the slope of the regression line on a plot of the log_culture turbidity_ versus time from at least 5 points during exponential phase. Coefficients of determination (R^2^) were >0.96 for each growth curve.

2) Survival rate in stationary phase of cultures on the indicated media was calculated as the ratio of the number of CFU/ml counted on MM+3CBA and the number of CFU/ml on MM+succinate plates. Succ, succinate; 3CBA, 3-chlorobenzoate.

3) Calculated standard deviation from triplicate measurements.

4) Calculated p-value in a two-tailed Student's t-Test using equal variance.

### *tciR* is a very widespread ortholog among ICE closely related to ICE*clc*

**Figure 7:**
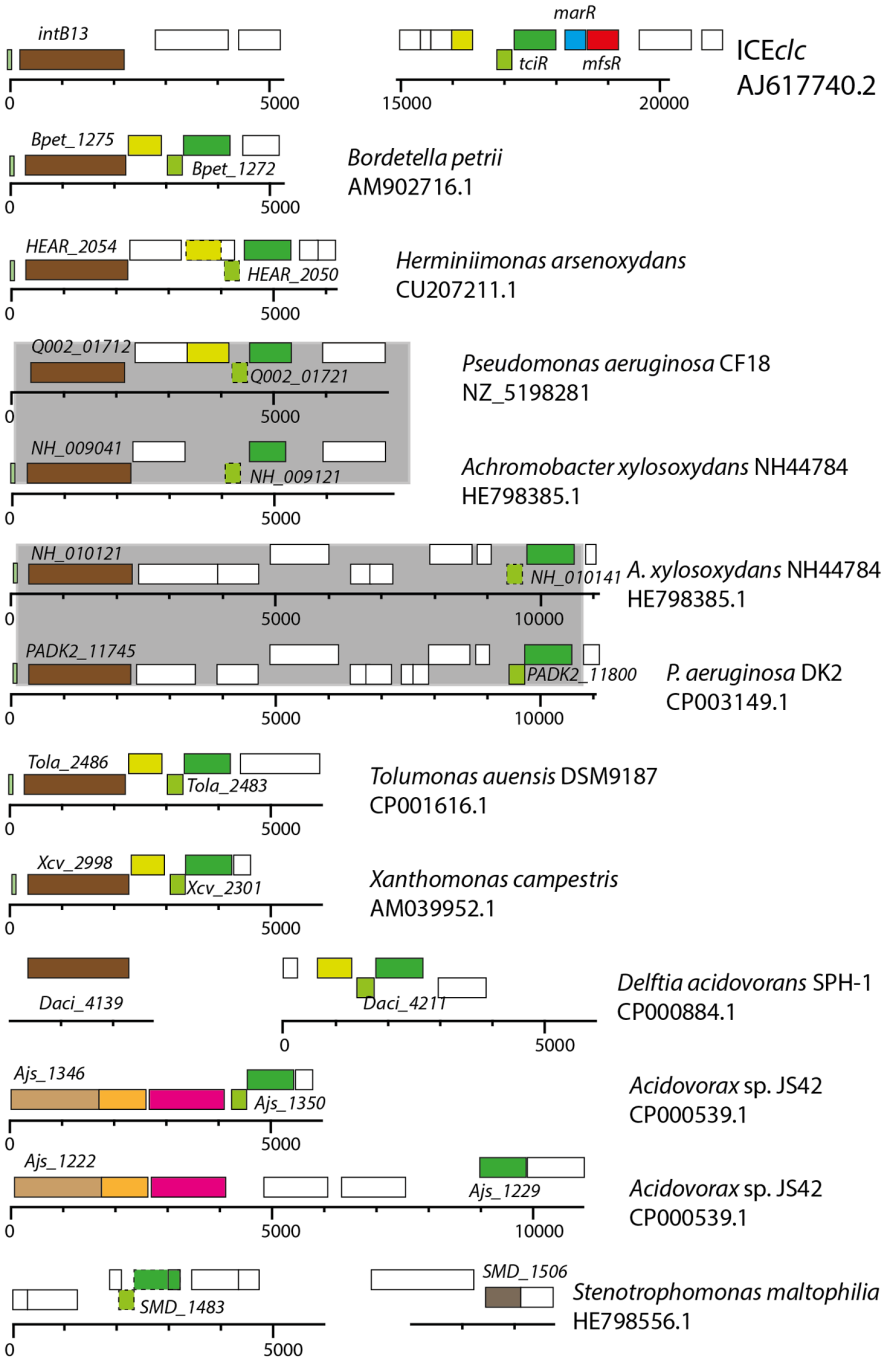
Conservation of *tciR* analogues in putative ICE*clc*-like regions in a variety of other bacterial genomes. Illustration represents *tciR* analogues (identified on the basis of a BLASTN E-value lower than 1·10^-15^), and surrounding relevant gene regions in the indicated bacterial genomes (species name, accession numbers) compared to ICE*clc*. Genes are indicated as in the respective genome accession. Rectangles show annotated genes and their orientation (top, orientation towards the left; bottom, gene orientation towards to right); common colors indicate similar predicted functions. Stippled rectangles indicate common gene regions inferred from Artemis comparison, but not present in the respective annotation. ICE were inferred from (i) more than 75% nucleotide identities across the complete core region of ICE*clc*, and within a 1-100 kb window from the *tciR* position, and (ii) the presence of an integrase gene (in brown) within a 5-20 kb window from the *tciR*-analogue. Note how some genomes carry multiple different ICE from the same family (e.g., *Achromobacter xylosoxidans*, *Acidovorax* sp. strain JS42), and further how pair-wise identical ICE regions (shaded in grey) occur between different genomes. Finally note how the *tciR*-analogues often co-occur with a *xer*-type regulatory gene on the other strand (light green), and a further *lysR* gene member (yellow), but in none of the cases shown here with an *mfsR* counterpart (in red).

Using bioinformatic queries, we retrieved orthologs to *tciR* and *mfsR* from sequenced bacterial genomes, and examined manually whether they occur in chromosomal regions qualifying as ICE (e.g., presence of an integrase gene nearby, see *Materials and Methods*). Interestingly, orthologs to the individual components of the *mfsR* operon are widespread but rarely occur in the same configuration (Figure 7). So far, the ICE*clc mfsR-marR-tciR* configuration is only found in the ICE*clc* variant of *Burkholderia xenovorans* LB400, whereas the Tn*4371*-element of *Acidovorax* sp. strain JS42 and *Aeromonas hydropohila* SSU (accession number AGWR01000022.1) both carry an *mfsR* homolog and a MarR-type regulator immediately downstream, but no *tciR* equivalent nearby. There is a *tciR* ortholog in *Acidovorax* sp. strain JS42, but not on the same chromosomal region as *mfsR* (Figure 7).

On the other hand, *tciR* seems much more widespread among ICEs, as homologs can be found in ICE*clc*-like elements GI1 and GI6 of *Bordetella petrii* DSM12804, in PAGI-2 of *P. aeruginosa* strain C, in diverse ICEs of *X. campestris* pv. vesicatoria str. 85-10*6*, suspected ICEs in *Herminiimonas arsenicoxydans*, *Cupriavidus metallidurans* CH34, and *Tolumonas auensis* DSM 9187, among several dozens of others (Figure 7). Given that TciR is such a common regulator found in ICEs of the ICE*clc* family, it might be similarly implicated in their transfer control. This regulation is likely different in detail from ICE*clc*, given the absence of an *mfsR* and *marR*.

## Discussion

**Figure 8:**
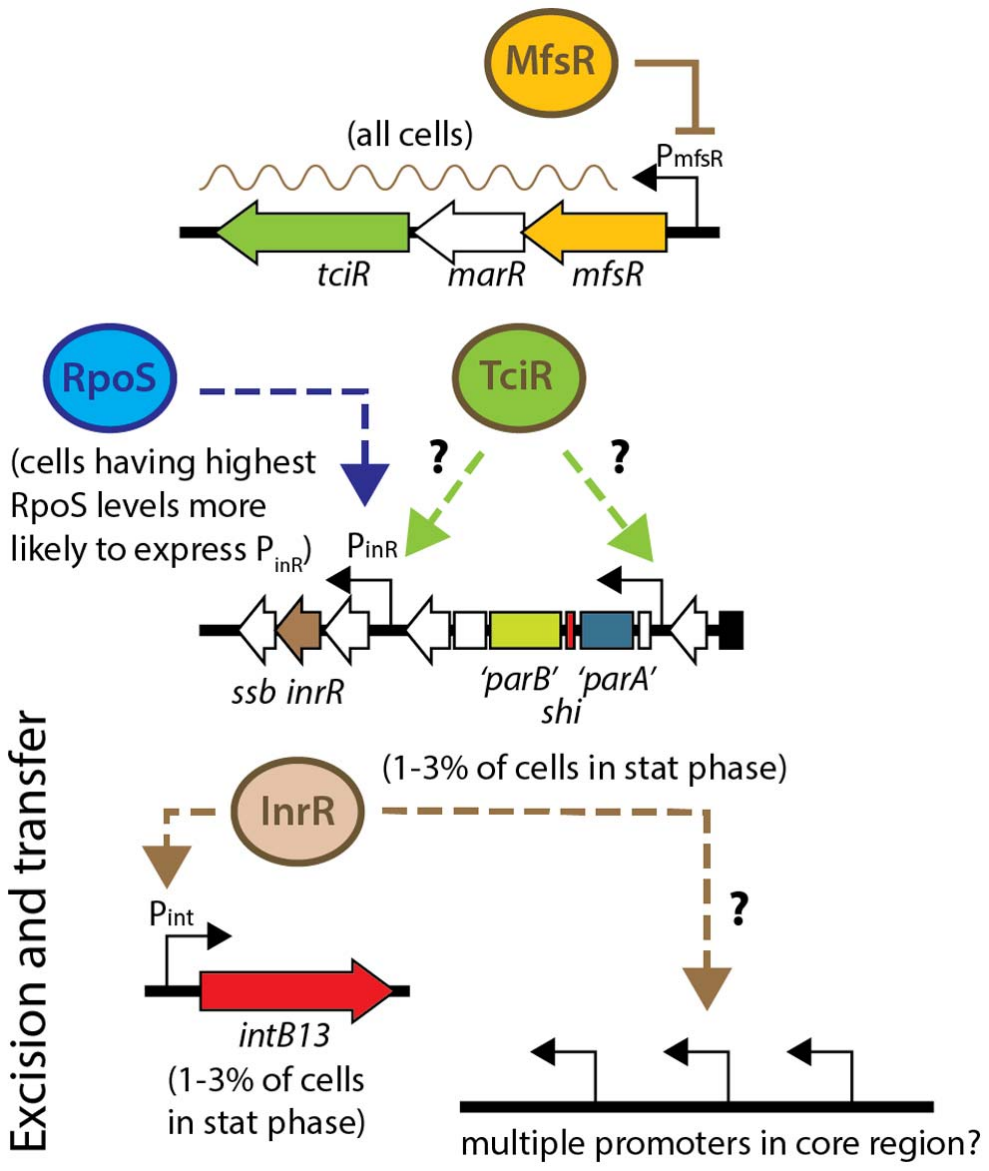
Model for regulation of ICE*clc* transfer competence. MfsR autoregulates expression of itself and of the TciR activator, without which ICE*clc* transfer decreases by 2·10^3^-fold. TciR may activate specifically one or more promoters on ICE*clc*, such as the RpoS-dependent P_inR_-promoter [29], or a promoter upstream of the *parA*-like gene [32]. Expression of the P_inR_-promoter occurs preferentially in cells having highest RpoS levels, and only 1-3% of cells in a population in stationary phase visibly express reporter gene from P_inR_ [29]. InrR transmits bistable activation through an unknown process to the *intB13* promoter [26], and possibly simultaneously to other promoters for the genes for the conjugative system in the ICE*clc* core region [32]. The exact mechanism of arisal of bistability is unclear as yet. For gene locations on ICE*clc*, see Figure 1A.

ICE*clc* has two distinctive modes of existence: the integrated form, which is transmitted vertically, and the circular form, which can be horizontally transferred. Previous work in our laboratory has shown that the transition between these two states occurs in only a few percent of cells in a population under stationary phase conditions [26], [30], [34]. We have recently suggested to name cells in which the molecular decision occurs to activate the ICE*clc* horizontal transfer mode *transfer competent* (tc) cells [30]. Single cell time-lapse experiments indicated that ICE*clc* transfer - at least insofar as detectable by microscopy, only occurs from tc cells, which can be distinguished through simultaneous expression of fluorescent proteins from single copy transcriptional fusions to the P_int_ and P_inR_ promoters of ICE*clc* [30]. Activation of those two promoters is the likely outcome of a multi-step regulatory cascade that orchestrates expression of some fifty genes [32], but the key factors that determine the onset of this cascade and control the extent of bistability are still obscure. Previous work provided evidence for the role of the stationary phase sigma factor RpoS in activation of ICE*clc* promoters, and we could show that tc cells on average have higher levels of RpoS [34]. In the present study, we report the discovery of a cluster of three regulatory genes, two of which globally control ICE*clc* activation and transfer, and additionally maintain a cap on fitness loss induced by the ICE (Figure 8).

The three regulatory genes occur in a unique configuration of a TetR-type repressor (encoded by *mfsR*), followed by a MarR-type (*orf17984*) and a LysR-type regulator (*tciR*). Transcript and microarray analysis of the locus in wild-type and mutant ICE*clc*, plus analysis of reporter gene expression from the *mfsR* promoter in a variety of host backgrounds, showed that the three genes are expressed as a polycistronic unit and are under autoregulatory control by MfsR (Figure 5A).

Precise gene deletions and complementations indicated that *tciR* is likely the main global regulator of ICE*clc* transfer activation. Deletion of *tciR* caused a 2·10^3^-fold lower frequency of ICE*clc* transfer compared to wild type (Figure 2B), silenced expression of the ICE*clc* core region (Figure 3B) and reduced the proportion of cells expressing P_int_ and P_inR_ in stationary phase (Figure 5B). Complementation of the *tciR* deletion on ICE*clc* with a single copy mini-Tn*7* inserted *mfsR^fs^-marR^fs^-tciR* fragment (Figure 1C) fused to the *mfsR* promoter region restored the expected phenotype (Figure 2B). TciR may act either directly as a regulator on a variety of individual ICE*clc* core promoters, or as a “master” regulator in a hierarchical activation cascade (Figure 8).

The role of *marR* is less clear and as yet unsolved. Deletion in *marR* resulted in essentially the same ICE*clc* transcriptome profile as deletion in *tciR* (Figure 3D). It also resulted in a lower transfer frequency than wild-type but not as low as the deletion in *tciR* (Figure 2B), and produced no detectable reporter gene expression from P_int_ or P_inR_ (Figure 5). In contrast, complementation of the *marR* deletion on ICE*clc* by a single copy *marR* gene through mini-Tn*7* delivery (Figure 1C) did not restore ICE*clc* transfer (Figure 2B). Furthermore, mutants with deletions in *mfsR* or *mfsR* plus the first 116 bp of *marR* (Figure 1B), behaved quite similar in transfer frequency (Figure 2) and showed similar ICE*clc* transcriptomes (Figure S4). The *marR* gene therefore seems to have no clear role in ICE*clc* core gene expression.

The most surprising effect of deletions in *mfsR* was a complete deregulation of the ICE*clc* core gene expression. This became obvious from frequencies of ICE*clc* transfer being 10-100 fold higher than wild type, approximating 1 transfer per donor cell (Figure 2B). The deregulation was also obvious in microarray data showing the ICE*clc* core region in the *mfsR* deletion mutants being already transcribed in exponential phase (Figure 3B, Figure S3). Finally, 80-100% of individual cells in stationary phase expressed the reporter genes from P_int_ and P_inR_ in the *mfsR* deletion strain compared to 3-5% in wild-type (Figure 5B). This can be explained by the fact that deletion of *mfsR* would abolish autorepression, which would lead to constant high expression of *marR* and *tciR.* This overinitiates ICE*clc* core gene expression, leads to more cells entering the tc state and to higher transfer rates. The balance of *mfsR* control appears to be extremely delicate, since even complementation with a single gene copy under control of the original promoter results in a stronger effect than the wild-type, both for *tciR* and *mfsR* (Figure 2B). The delicate balance became also obvious from the polar effects of insertion of the Km-resistance gene within *mfsR*, leading to decreased transcription of *marR* and *tciR*, diminished core gene expression (Figure 3D) and reduced transfer rates (Figure 2A).

The finding that deletion and complementation of *mfsR* or *tciR* drastically changes the proportion of cells activating ICE*clc,* could imply that the bistability seen in wild-type situation (i.e., 3-5% of cells in stationary phase becoming transfer competent) is a result of feedback at this locus. Cells activating ICE*clc* in the wild-type situation could arise as a consequence of “sloppy” control by MfsR, incidentally causing a few cells to escape its control and transcribing *marR* and *tciR*. We think this is an unlikely scenario, because mCherry expression from the *mfsR* promoter is homogenous among cells (Figure 5A). Alternatively, there might be a chemical ligand that specifically binds to MfsR in a small subset of cells, upon which its repression is relieved in those cells. The resulting TciR would then be the necessary activator to trigger ICE*clc* core expression in cells with on average highest RpoS levels [29] (Figure 8). On the other hand, even though *mfsR* may be the first level of control, bistability may also originate at later checkpoints in the regulatory cascade, which depend on the presence of sufficient TciR.

Quasi-global appearance of transfer competence across all cells in the *mfsR* deletion mutant resulted in massive fitness loss (Figure 6), which became evident at two levels. First of all, time-lapse observations indicated lysis and aberrant cell growth in more than 50% of microcolonies (Figure 6B). This lysis and growth arrest are similar to what we previously described as being a side consequence of becoming transfer competent in wild-type cells [30], and is caused by the *parA-shi* gene products on ICE*clc* [30]. Secondly, there was a strong loss of the capacity to grow on 3CBA among cells sampled from stationary phase cultures of the *mfsR* mutant compared to wild-type (Figure 6A, Figure S6), indicative for loss of ICE*clc* and counterselection against maintaining ICE*clc-*Δ*mfsR*. However, those cells that maintained ICE*clc-*Δ*mfsR* could still grow on 3CBA and showed indistinguishable exponential growth rate (Table 2). This paradox can be understood when modeling the number of tc cells in batch culture populations for ICE*clc* wild-type (probability of tc arisal, *P*_tc_, in stationary phase of 0.025) and for the ICE*clc*-Δ*mfsR* mutant (*P*_tc_ = 0.5). This model (Figure S5B) shows that whereas a large proportion of tc cells appear in ICE*clc*-Δ*mfsR* mutant cultures in stationary phase, these can only divide 2-3 times upon reinoculation into fresh medium before lysing. This causes an apparent prolongation of a lag phase visible as stagnant culture turbidity, but does not influence the overall predicted population exponential growth rate in batch culture (Figure S5B).

As expected from the postulated role of TciR, its complementation *in trans* also leads to increased ICE*clc* transfer, but interestingly, only the *mfsR* deletion caused strongly decreased cell survival (Figure S6). We therefore hypothesize that ICE*clc* activation may follow two separate processes: transfer and tc cell growth arrest [30], that may both be initiated at the *mfsR* locus. Deleting *mfsR* would then deregulate both processes, whereas expressing *tciR* in trans would only increase activation through the transfer branch (Figure 8).

The configuration of the *mfsR-marR-tciR* operon of ICE*clc* is unique, but *tciR* alone is a very common part of ICE similar to ICE*clc* (Figure 7). We therefore speculate that *mfsR-marR* are a more recent acquisition in ICE*clc*, which drastically changed the expression of the *tciR* gene. Unfortunately, expression of *tciR* analogs in other ICEs has not been studied and very little has been reported on the transferability of ICEs related to ICE*clc*. The exceptions being GI3 of *B. petrii* that transfers at extremely low frequencies (∼10^-7^) [35], and the *P. aeruginosa* PAGI-2 element for which transfer has not been detected at all [16]. In comparison, wild-type ICE*clc* transfers at rates of 10^-2^ to 10^-3^ per donor (Figure 2), suggesting that it was perhaps the acquisition of the *mfsR* regulatory control that led to expression of transfer activity in a larger proportion of cells in the population. As we show here, the downside of increasing the proportion of ICE*clc* tc cells is an increase of the proportion of cells displaying growth arrest through the *shi-parA* pathway [30]. Likely, the MfsR autoregulation evolved to a stage of permitting efficient transfer but avoiding too much fitness loss to the population. Even though the mechanistic details are different for ICE*clc*, double control layers are more common for various ICEs and typically involve a variety of regulators acting on each other and/or in response to specific chemical ligands [18], [19], [23]-[25]. It will be highly interesting to further study the mechanistic details of the control systems that maintain very low ICE transfer rates, and to understand whether and how such control can evolve to allow hyperefficient transfer.

## Materials and Methods

### Strains and culture conditions

Table 1 lists the strains used in this study. *Escherichia coli* DH5α (Gibco Life Technologies, Gaithersburg, Md.), *E. coli* DH5α λpir, *E. coli* BW20767/pRL27 were cultured at 37°C on Luria-Bertani (LB) medium [36]. *Pseudomonas* species were cultured at 30°C on LB or 21C minimal medium (MM) [37] complemented with one of the following carbon sources: 0.5, 5, or 10 mM 3-chlorobenzoate (3CBA), 15 mM succinate or 10 mM fructose. Antibiotics were supplemented to the growth medium to select for maintenance of genetic constructions at the following concentrations: kanamycin (Km) 25 µg/ml, chloramphenicol (Cm) 20 µg/ml, rifampicin (Rif) 50 µg/ml, nalidixic acid (Nal) 50 µg/ml, gentamicin (Gm) 20 µg/ml, and ampicillin (Ap) 100 µg/ml.

### Strain constructions and DNA techniques

DNA purification, PCR, restriction enzyme digestions, DNA ligations and electro-transformations were performed according to standard procedures [36]. Deletions in ICE*clc* genes were created by double recombination techniques as described elsewhere [29], [38]. Nucleotide positions are given according to AJ617740 (ICE*clc*). Primers used for strain constructions are listed in table S1.

For complementation of *P. putida* UWC1 (ICE*clc*-Δ*mfsR*, strain 4322) we first amplified the *mfsR* gene plus the 429 bp upstream region containing the *mfsR* promoter using PCR. This fragment was cloned into pGEM-T-easy and verified for correctness by DNA sequencing. The fragment containing the correct *mfsR* region was then recovered by restriction enzyme digestion with PstI and BamHI, and ligated into the mini-Tn*7* vector pUC-miniTn*7*-Gm [39]. After transformation and verification in *E. coli*, the mini-Tn*7* construct was introduced into *P. putida* UWC1 (ICE*clc*-Δ*mfsR*) by using the pUX-BF13 helper plasmid [40]. Clones resistant to Gm were selected and verified by PCR for correct insertion of the *mfsR* DNA in the *attTn*7 locus. To complement *P. putida* UWC1 (ICE*clc*-Δ*tciR*, strain 4321) and (ICE*clc*-Δ*marR*, strain 4372) we amplified the complete *mfsR-marR-tciR* locus including the 429-bp upstream region. This fragment was cloned into pGEM-T-easy and again verified for correctness by DNA sequencing. The fragment was recovered by digestion with BamHI and StuI, and ligated with the mini-Tn*7* vector. A frameshift was then introduced in the *mfsR* coding region by digestion at the unique NcoI-site, filling in using Klenow and religation. This will cause premature ending of the *mfsR* gene product (*mfsR^fs^)*. A second frameshift was subsequently introduced to inactivate the *marR* gene product, using the unique BsgI-site (*marR^fs^)*. After transformation and verification in *E. coli*, the construct was introduced in *P. putida* UWC1 (ICE*clc*-Δ*tciR*) as outlined above. This procedure was repeated to create a fragment with frameshifts in *mfsR* and in *tciR* (using the unique KpnI site), but maintaining an intact *marR*. This construct was introduced into *P. putida* UWC1 (ICE*clc*-Δ*marR*). Gm-resistant clones were verified by PCR for the correct insertion at the *attTn*7-site, and for the presence of ICE*clc*.

A 656-bp region upstream of *mfsR* was amplified by PCR and fused to a promoterless *mcherry* gene. This fragment was introduced in single copy on the chromosome of *P. putida* UWC1, *P. putida* UWC1 (ICE*clc*) or *P. putida* UWC1 (ICE*clc*-Δ*mfsR*) using mini-Tn*5* delivery. Three independent Km-resistant colonies were verified by PCR for the correct insertion and stored individually. *P. putida* UWC1 mini-Tn*5*-P_mfsR_-*mcherry* and *P. putida* UWC1 (ICE*clc*-Δ*mfsR*) mini-Tn*5-*P_mfsR_ *-mcherry* were then further used as recipient to introduce the mini-Tn*7*-*mfsR* construct.

### Random mutagenesis and screening

Random mini-transposon insertions in *P. knackmussii* B13 were generated by mobilization of the pRL27 suicide plasmid from *E. coli* BW20767 in a biparental mating. Hereto both strains were each cultured overnight in 3 ml LB, pelleted down, resuspended in 50 µl sterile saline solution (0.9% NaCl), mixed in a 1∶1 (*v/v*) ratio and incubated on the surface of an LB agar plate for 24 hours at 30°C. The mixture was then resuspended with 1 ml saline solution, which was inoculated in 100 ml MM with 0.5 mM 3CBA plus Km to select for the mini-transposon insertion and Cm to counterselect against *E. coli*, and incubated at 30°C for 16 h with orbital shaking (180 rpm). An aliquot of 3 ml of this pool of enriched Km^R^ B13 mutants was used *en masse* as donor in a subsequent mating procedure. Hereto, cells from the 3 ml suspension were pelleted by centrifugation, washed with 3 ml sterile saline and mixed with 3 ml of suspension of *P. putida* UWC1 recipient, that had been grown for 16 h on LB, was pelleted by centrifugation and resuspended in sterile saline. The mating mixture was again centrifuged, the cell pellet was resuspended in 50 µl sterile saline solution and spotted on the surface of a MM agar plate containing 0.5 mM 3CBA. The mixture was incubated for 72 hours at 30°C, after which the cells were washed from the plate with 1 ml sterile saline, which was further serially diluted and plated on MM agar plates with 5 mM 3CBA plus Km and Rif to select for transconjugants carrying mutant ICE*clc*. Individual colonies were purified, recultured in organized 96-well format and stored at -80°C after addition of and mixing with glycerol to 15% (*v/v*). Libraries were replicated and regrown in 100 µl LB plus Rif for 16 h in 96-well microtiter plates, mixed with 100 µl *P. putida* UWC1 Nal^R^ recipient suspension, and incubated at 30°C for 48 h. Then 50 µl of each well was reinoculated into 170 µl of MM containing 5 mM 3CBA plus Km, Rif and Nal, and growth was measured by continuous OD-measurements in a multiplate reader (FluoStar Omega, BMG labtech). Absence of growth was taken as indication for absence of ICE*clc* transfer, in which case the donor culture was recovered for mapping of the transposon insertion.

### Insertion mappings

DIG-labeled primers 070934 or 070935, annealing to one of the ends of the Km^R^ insert but facing outward, were used (separately) in single-primer PCR with DNA from mutant UWC1 donors as templates. The reactions produced oligonucleotide probes with the 5′-DIG label, the sequence of the end of the Km^R^ gene and the adjacent sequence of the ICE*clc* insert position. Such products were used for rough localization of the insertion position by hybridizing to macroblot membranes (Eurogentec, UK), whose set of 55-mer oligonucleotides covers most of the ICE*clc* genes. Hybridization and detection of the DIG-marker were carried out according to the manufacturer's instructions (Roche Diagnostics GmbH, Mannheim, Germany). Once the insertion was roughly mapped on ICE*clc*, PCR-based sequencing was used to determine exact position of the Km^R^-gene insertion.

### ICE*clc* transfer assays

The frequency of ICE*clc* transfer was determined in experimental conditions described previously [33]. *P. putida* UWC1 ICE*clc* wild-type or mutant derivatives were used as donors, whereas *P. putida* UWCGC (constitutively fluorescent, Gm^R^) or *P. putida* UWC1 Km^R^ were used as the recipient (Table 1). Briefly, donors and recipient were each cultivated on 5 mM 3CBA MM and 10 mM fructose MM, respectively, and combined on 0.5 mM 3CBA agar plates as a single concentrated pellet. After 48 hours incubation at 30°C, mating mixes were resuspended, diluted and plated on 5 mM 3CBA MM agar (counting of donor CFU) or 5 mM 3CBA Gm or Km agar (counting of transconjugant CFU). Transconjugants were checked by PCR and frequencies were expressed as the number of transconjugant CFU per donor CFU. Donor survival was used for the data shown in Figure S6.

### ICE*clc* transcriptome analysis by microarrays

The ICE*clc* transcriptomes of *P. putida* UWC1 (ICE*clc*), *P. putida* UWC1 (ICE*clc*), *P. putida* UWC1 (ICE*clc*-Km^R^_19033_), *P. putida* UWC1 (ICE*clc*-Δ*'marR*), *P. putida* UWC1 (ICE*clc*-Δ*'tciR*), *P. putida* UWC1 (ICE*clc*-Δ*mfsR-*Δ*'marR*), *P. putida* UWC1 (ICE*clc*-Δ*mfsR*) were investigated by microarray analysis, as described previously [32]. Total RNA was extracted from cells grown on 10 mM 3CBA MM, and harvested at mid exponential phase (OD_600_ = 0.6) and 48 h after entrance in stationary phase. Reverse transcription using cyanine-dCTP among the dNTPs produced labeled cDNA that was further purified and hybridized on 8×15 K microarray slides (Agilent, Santa Clara, CA, USA). Slides were washed and scanned according to manufacturer's instructions (Agilent). Data were recovered and analyzed using GeneSpring GX. Microarray data can be accessed from the GEO database (accession number: GSE51391).

### Time-lapse microscopy

*P. putida* UWC1 strains were precultured for 16 h at 30°C in LB medium, after which 100 µl were transferred to 20 ml fresh MM 4 mM 3CBA medium in presence of the appropriate antibiotics. This culture was incubated for 96 hours at 30°C and 200 rpm shaking, after which the cells were 100-fold diluted in MM without C-source and inoculated on agarose surfaces (*gel patches*) for time-lapse microscopy [41]. Medium for gel patches consisted of 1% agarose dissolved by heating into MM with 0.1 mM 3CBA. Gel patches were created by pipetting 130 µl of the agarose-MM-3CBA solution kept at 55 °C on the surface of a circular cover glass (42 mm ø and 0.17-mm thick), placed in an autoclaved perfusion chamber (POC, H. Saur, Reutlingen, Germany), separated with a 0.5 mm thick silicon spacer ring and covering them with a second cover glass. After solidification of the agarose, the upper cover slip was removed and 6 µl of the diluted cell suspension was placed onto the agarose gel patch. As soon as the drops were dried on the surface, the patches were turned upside down and placed bacteria-facing-down on a new round cover glass [41]. A second silicon spacer ring was added to allow air circulation within the closed chamber and the glass sandwich was fixed into the metal cast POC chamber with a metal ring. Up to four patches could be placed simultaneously within a single glass sandwich in a POC chamber.

Microcolony development was followed directly on a Nikon Inverted Microscope Eclipse Ti-E, equipped with a Perfect Focus System (PFS), pE-100 CoolLED and a Plan Apo λ 100×1.45 Oil objective (Nikon), installed in a controlled temperature room (22°C). Ten random regions of every patch were imaged automatically during 48 hours with intervals of 1 h, in Phase Contrast mode (10 ms exposure), eGFP (500 ms) and eCherry (500 ms). Images were recorded using Micro-Manager 1.4 (http://www.micro-manager.org/) and fluorescence values were extracted using MetaMorph (Series 7.5, MDS, Analytical Technologies).

### *P. putida* UWC1 ICE*clc* and mutant fitness tests

Triplicates of strains UWC1 (ICE*clc*) and UWC1 (ICE*clc*-Δ*mfsR*) were grown for 16 h in LB medium at 30°C. Both strains were then 500-fold diluted (starting OD_600_ 0.001) in MM with 5 mM 3CBA or 10 mM succinate. Upon reaching early stationary phase, strains were again diluted into fresh MM (starting OD 0.001) with the same carbon source, and growth was followed by frequent culture turbidity measurements (OD_600_). 24 h after reaching stationary phase, each replicate culture was serially diluted in MM and plated onto MM agar plates with 5 mM 3CBA or with 10 mM succinate. The number of CFU/ml was scored and the ratio was calculated between the number of CFU/ml on MM agar with 5 mM 3CBA and the number of CFU/ml on MM with 10 mM succinate.

Ten randomly chosen colonies of UWC1 (ICE*clc*-Δ*mfsR*) cultivated in MM with succinate and grown on MM-succinate agar plates were retested for growth on MM agar with 5 mM 3CBA. The presence of ICE*clc* was determined by colony PCR on the same colonies by amplifying the *clcA* gene, which is carried by ICE*clc* and the gene product of which is essential for 3CBA metabolism.

### Bioinformatic screening for ICE related to ICE*clc*

Homologues to *tciR* of ICE*clc* were detected by BLASTN to the nr/nt database at E-value <1·10^-15^. The corresponding whole or draft genome sequences were retrieved and compaired by aligning to ICE*clc* (Accession number AJ617740.2) using Megablast. Detected regions were manually recovered and searched for the *tciR* homologue and an *intB13* homologue within a 1-100 kb window. If annotated, the presence of a gene for tRNA-Gly nearby the *intB13* homologue was scored. Regions covering all criteria (i.e., homology to ICE*clc* core region, *tciR* homologue and presence of integrase gene) were retained as containing putative ICE. Selected regions were further individually pair-wise compared by using the Artemis Comparison Tool within the WebACT service [42].

## Supporting Information

Figure S1 Outline of the random mutagenesis and subsequent selection procedure. Original ICE*clc*-host *P. knackmussii* B13 is randomly mutagenized by miniTn5-mediated insertions of kanamycin resistance inserts (Km^R^). Mutant B13 are selected by culturing cells on minimal medium (MM) with Km and 3-chlorocatechol (3CBA) as sole carbon and energy source. The pool of B13 mutants is cultured in batch and mixed with recipient strain *P. putida* UWC1 (resistant to rifampicin, Rif^R^). The mixture is incubated in mating conditions for 72 hours and plated on MM with 3CBA, Km and Rif, to select for transconjugants. Individual colonies of transconjugants were restriked and organized into a mutant library in 96-well plates. Each mutant is used as donor in a new 96-well mating with recipient *P. putida* UWC1 resistant to Rif and nalidixic acid (Nal^R^). Individual mating mixtures are grown on MM agar with 3CBA, Km, Rif, Nal in order to select for transconjugants. In absence of transconjugant growth, the donor of that particular mating was recovered from the library and had its insertion position mapped.
(TIF)

Figure S2 Reverse transcriptase polymerase chain reaction analysis of transcription in the *mfsR-marR-tciR* region. (A) Amplification of specific regions on reverse-transcribed (+) or not (-) mRNA purified from exponentially growing *P. putida* UWC1 (ICE*clc*) cultures on 3CBA, compared to amplification on purified DNA. (B) Schematic overview of the location of the used primers for the reverse transcription reaction (RT) and for the amplification of the gene regions.
(TIF)

Figure S3 Pair-wise comparisons of expression in the ICE*clc* area by microarray analysis. (A) *P. putida* UWC1 (ICE*clc*-Δ*marR*, strain 4372) compared to *P. putida* UWC1 (ICE*clc*, strain 2737). (B) *P. putida* UWC1 (ICE*clc*-Δ*tciR*, strain 4321) versus *P. putida* UWC1 (ICE*clc*, strain 2737). (C) *P. putida* UWC1 (ICE*clc*-Δ*marR*, strain 4372) versus *P. putida* UWC1 (ICE*clc*-Δ*tciR*, strain 4321). Panels indicate comparisons of exponentially growing or stationary phase cells, with hybridization signals on the plus- (open symbols) or minus-strand (closed symbols) of ICE*clc*. Dots indicate the ^2^log-fold change of hybridization signal per microarray probe in the comparison, plotted at their distance along the ICE*clc* sequence (X-axis; in kb). A scheme of ICE*clc* is redrawn at the bottom of each section, with regions of interest as grey boxes (+ or - indicate the DNA strand on which the region is encoded). Grey bars in the background indicate the two-fold cut-off level.
(TIF)

Figure S4 Pair-wise comparisons of expression in the ICE*clc* area of *mfsR* mutants by microarray analysis. (A) *P. putida* UWC1 (ICE*clc*-Δ*mfsR*, strain 4322) compared to *P. putida* UWC1 (ICE*clc*, strain 2737). (B) *P. putida* UWC1 (ICE*clc*-Δ*mfsR*-Δ'*marR*, strain 3453) versus *P. putida* UWC1 (ICE*clc*, strain 2737). (C) *P. putida* UWC1 (ICE*clc*-*mfsR::Km^R^*, strain 2961) versus *P. putida* UWC1 (ICE*clc*, strain 2737). Panels indicate comparisons of exponentially growing or stationary phase cells, with hybridization signals on the plus- (open symbols) or minus-strand (closed symbols) of ICE*clc*. Dots indicate the ^2^log-fold change of hybridization signal per microarray probe in the comparison, plotted at their distance along the ICE*clc* sequence (X-axis; in kb). A scheme of ICE*clc* is redrawn at the bottom of each section, with regions of interest as grey boxes (+ or - indicate the DNA strand on which the region is encoded). Grey bars in the background indicate the two-fold cut-off level.
(TIF)

Figure S5 Observed and expected population growth in cultures of *P. putida* with wild-type ICE*clc* and the Δ*mfsR* mutant. (A) Measured turbidities in triplicate batch cultures growing on 3CBA as sole carbon and energy source. Note the increased lag time of the *P. putida* Δ*mfsR* mutant. (B) Modeled population growth of all cells (brown) and tc cells (red) in two subsequent batch cultures growing on 3CBA; the second being inoculated with 1∶1000 volume from the first culture in stationary phase. Scenarios show predicted behavior for wild-type ICE*clc* (with a probability *P*_tc_ of 0.025 of tc cell appearance in stationary phase) and for Δ*mfsR* (with *P*_tc_ = 0.5). Note how a tc population appears in stationary phase, which is transferred to a new culture, but rapidly dies as a result of activation of the *par-shi* system [30]. Note further how exponential growth rates remain the same for both wild-type ICE*clc* and the Δ*mfsR* mutant. Modeling based on Monod kinetics, using the following parameters: total C = 4.25 mg C/ml, dry weight of a single cell = 2·10^-12^ g, yield = 0.3 g/g, K_s_ = 0.02 mg/mL, generation time of non-tc cells = 1 h, generation time of tc-cells = 2 h, death rate of tc cells = 0.4 h^-1^.
(TIF)

Figure S6 Population sizes of *P. putida* UWC1 carrying wild-type (2737, 2738) or mutant ICE*clc*, measured as colony forming units (CFU) on 5 mM 3-chlorobenzoate agar plates, per ml of resuspended culture spotted and incubated for 48 h on 0.5 mM 3-chlorobenzoate containing agar medium. (A) *P. putida* UWC1 strains: 4165, ICE*clc* with a deletion in the genes for the suspected efflux system (as unrelated control); 4321, *tciR* deletion; 4322, *mfsR* deletion; 4372, *marR* deletion; versus *P. putida* UWC1 (ICE*clc*), 2738. (B) *P. putida* UWC1 strains: 2737, ICE*clc* wild-type; 4321, *tciR* deletion; 4322, *mfsR* deletion; 4372, *marR* deletion; 4646, *mfsR* deletion but complemented *in trans* by a single copy mini-Tn*7* inserted *mfsR* gene under its own promoter; 4649, *tciR* deletion but complemented *in trans* by a single copy mini-Tn*7* inserted fragment with the (*mfsR^fs^)-(marR^fs^)-tciR* genes under the *mfsR* promoter. 4804, *marR* deletion but complemented *in trans* by a single copy mini-Tn*7* inserted fragment with the (*mfsR^fs^)-marR-*(*tciR^fs^*) genes under the *mfsR* promoter. (A) and (B), Independently carried out experiments on different occasions and by different scientists. Letters indicate statistically indistinguishable groups identified from ANOVA tests on biological replicates, followed by Tukey's post hoc testing. P-values indicate the significance for the overall group difference (*a* versus *b*), or in one specific case, between the samples connected by the line.
(TIF)

Table S1 Oligonucleotides used for amplification of ICE*clc* fragments.
(DOCX)
