# Correction: An Operon of Three Transcriptional Regulators Controls Horizontal Gene Transfer of the Integrative and Conjugative Element ICE*clc* in *Pseudomonas knackmussii* B13

**DOI:** 10.1371/journal.pgen.1005130

**Published:** 2015-04-10

**Authors:** The PLOS Genetics Staff

The accession number provided in the first paragraph of the Results sections, AJ617440.2, is incorrect. The correct accession number is AJ617740.2.
