# Supplementary figures and images for: An Operon of Three Transcriptional Regulators Controls Horizontal Gene Transfer of the Integrative and Conjugative Element ICEclc in Pseudomonas knackmussii B13
Source: PLoS Genet. 2014 Jun 19;10(6):e1004441. doi: 10.1371/journal.pgen.1004441 (PMC4063739; doi:10.1371/journal.pgen.1004441)

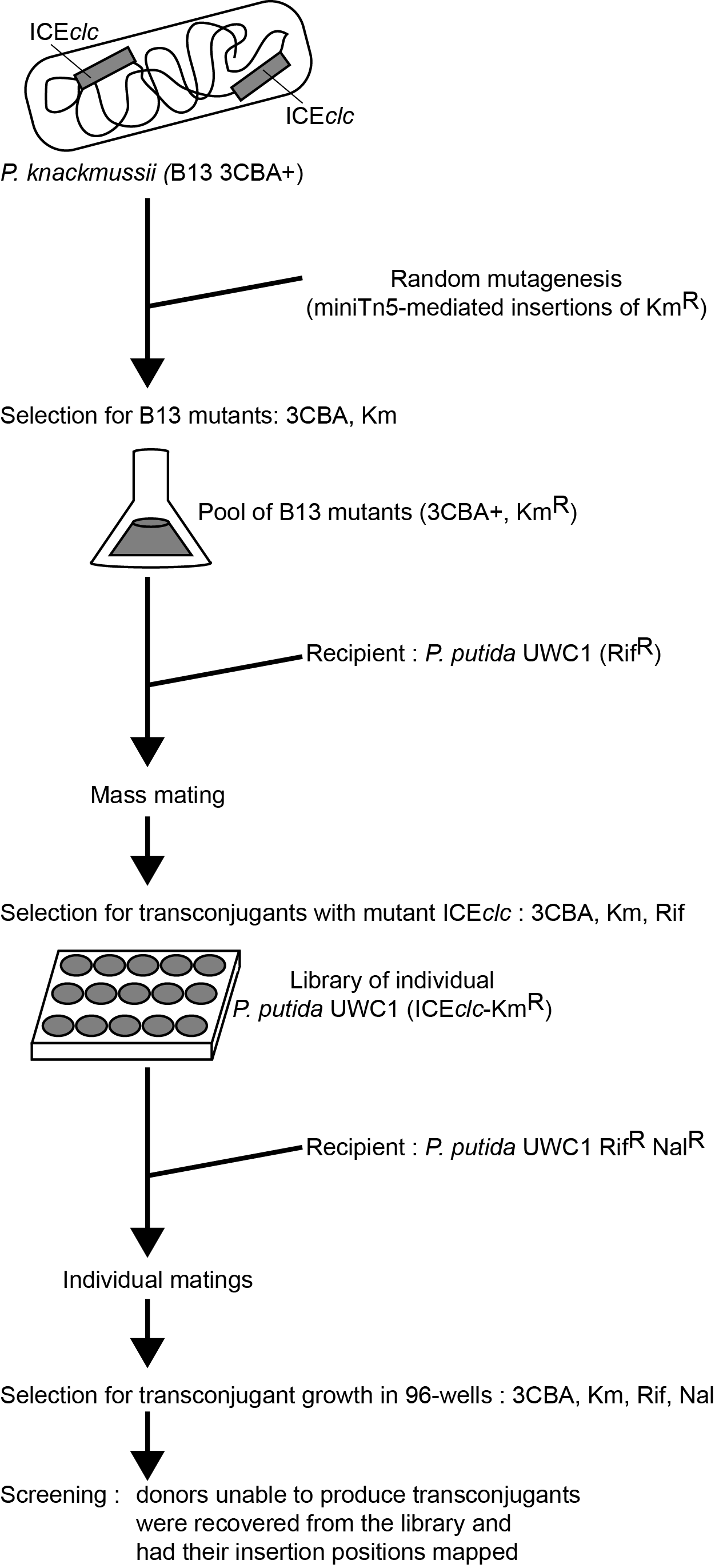

Supplement: Figure S1 — Outline of the random mutagenesis and subsequent selection procedure. Original ICEclc-host P. knackmussii B13 is randomly mutagenized by miniTn5-mediated insertions of kanamycin resistance inserts (KmR). Mutant B13 are selected by culturing cells on minimal medium (MM) with Km and 3-chlorocatechol (3CBA) as sole carbon and energy source. The pool of B13 mutants is cultured in batch and mixed with recipient strain P. putida UWC1 (resistant to rifampicin, RifR). The mixture is incubated in mating conditions for 72 hours and plated on MM with 3CBA, Km and Rif, to select for transconjugants. Individual colonies of transconjugants were restriked and organized into a mutant library in 96-well plates. Each mutant is used as donor in a new 96-well mating with recipient P. putida UWC1 resistant to Rif and nalidixic acid (NalR). Individual mating mixtures are grown on MM agar with 3CBA, Km, Rif, Nal in order to select for transconjugants. In absence of transconjugant growth, the donor of that particular mating was recovered from the library and had its insertion position mapped. (TIF) [file pgen.1004441.s001.tif]

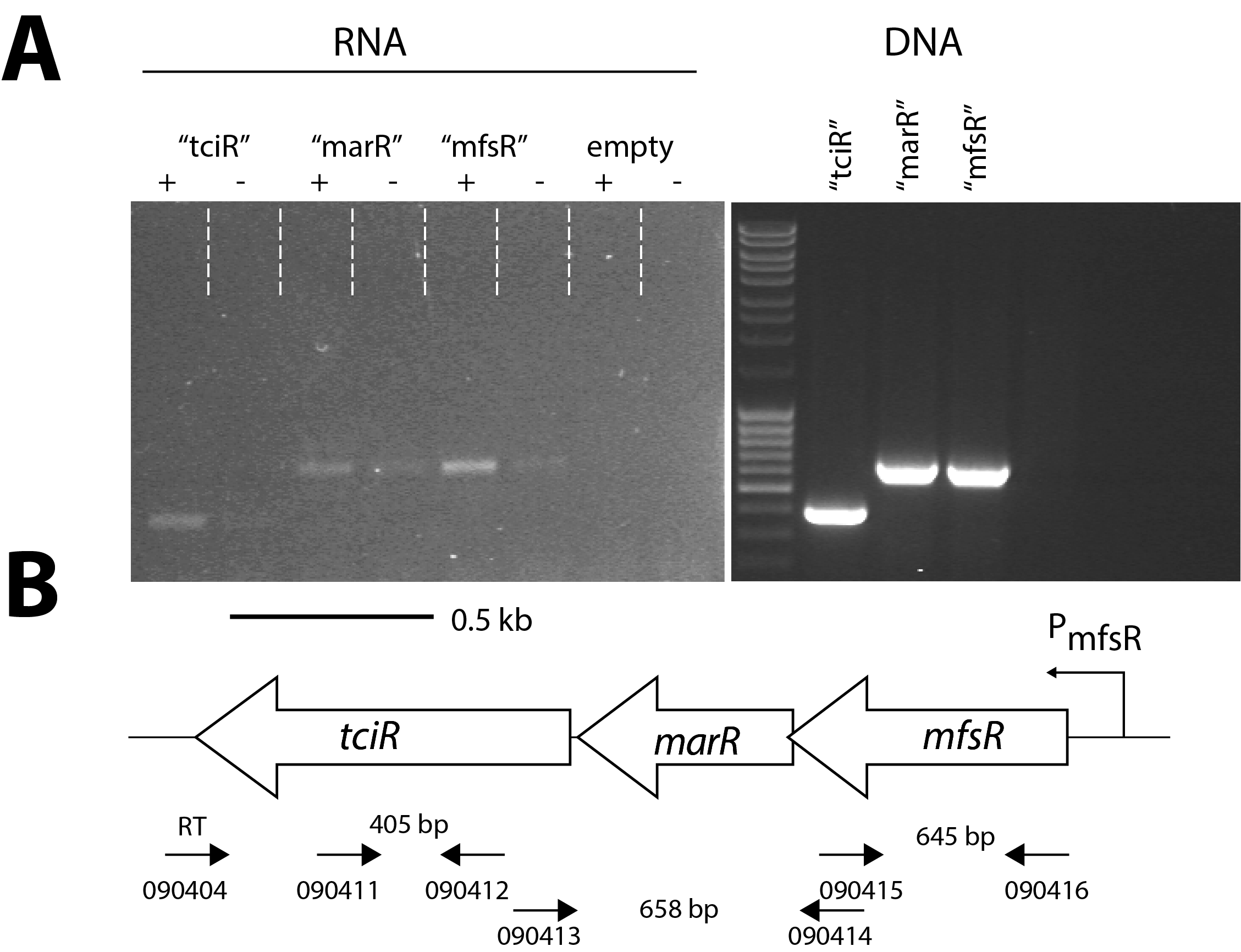

Supplement: Figure S2 — Reverse transcriptase polymerase chain reaction analysis of transcription in the mfsR-marR-tciR region. (A) Amplification of specific regions on reverse-transcribed (+) or not (-) mRNA purified from exponentially growing P. putida UWC1 (ICEclc) cultures on 3CBA, compared to amplification on purified DNA. (B) Schematic overview of the location of the used primers for the reverse transcription reaction (RT) and for the amplification of the gene regions. (TIF) [file pgen.1004441.s002.tif]

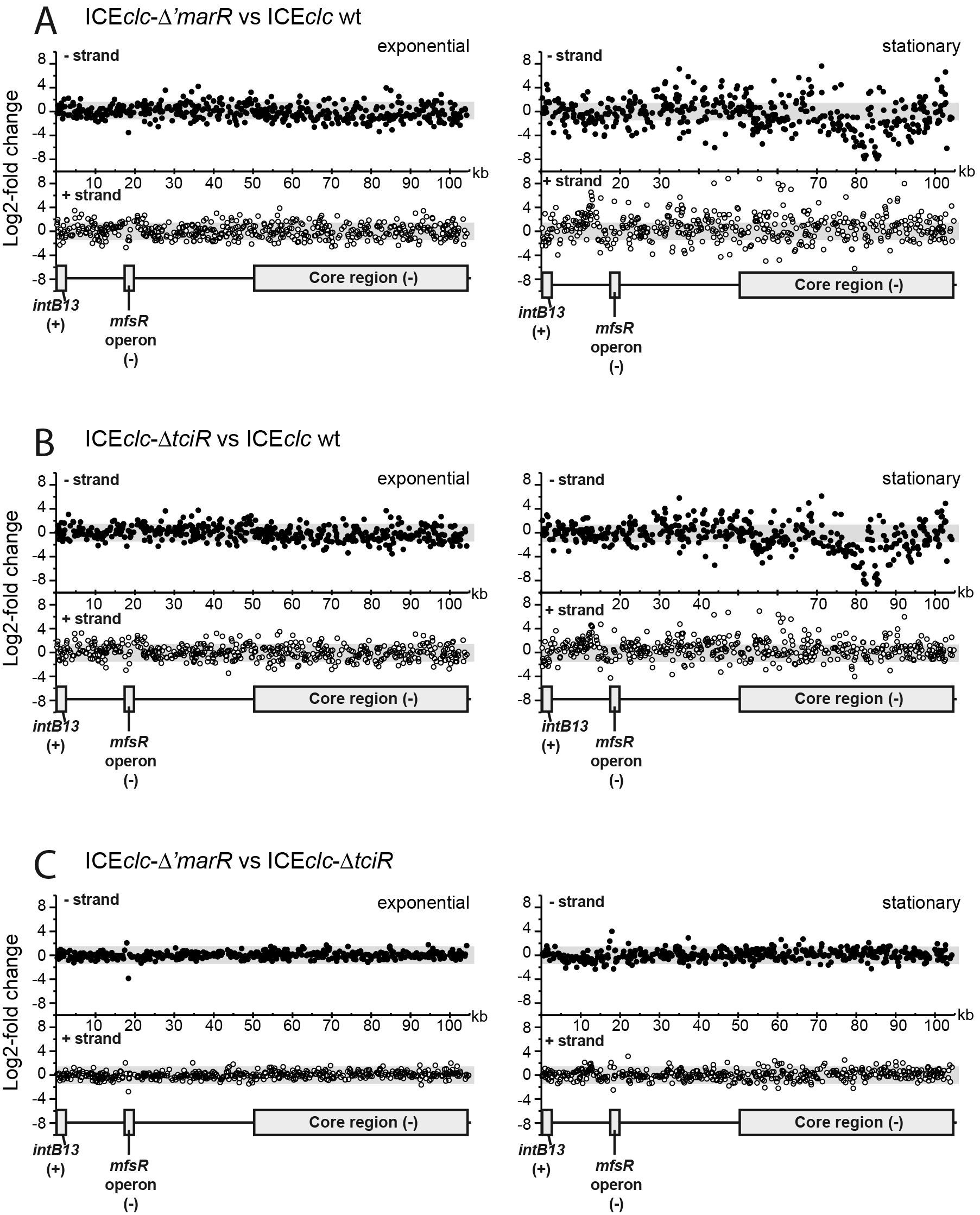

Supplement: Figure S3 — Pair-wise comparisons of expression in the ICEclc area by microarray analysis. (A) P. putida UWC1 (ICEclc-ΔmarR, strain 4372) compared to P. putida UWC1 (ICEclc, strain 2737). (B) P. putida UWC1 (ICEclc-ΔtciR, strain 4321) versus P. putida UWC1 (ICEclc, strain 2737). (C) P. putida UWC1 (ICEclc-ΔmarR, strain 4372) versus P. putida UWC1 (ICEclc-ΔtciR, strain 4321). Panels indicate comparisons of exponentially growing or stationary phase cells, with hybridization signals on the plus- (open symbols) or minus-strand (closed symbols) of ICEclc. Dots indicate the 2log-fold change of hybridization signal per microarray probe in the comparison, plotted at their distance along the ICEclc sequence (X-axis; in kb). A scheme of ICEclc is redrawn at the bottom of each section, with regions of interest as grey boxes (+ or - indicate the DNA strand on which the region is encoded). Grey bars in the background indicate the two-fold cut-off level. (TIF) [file pgen.1004441.s003.tif]

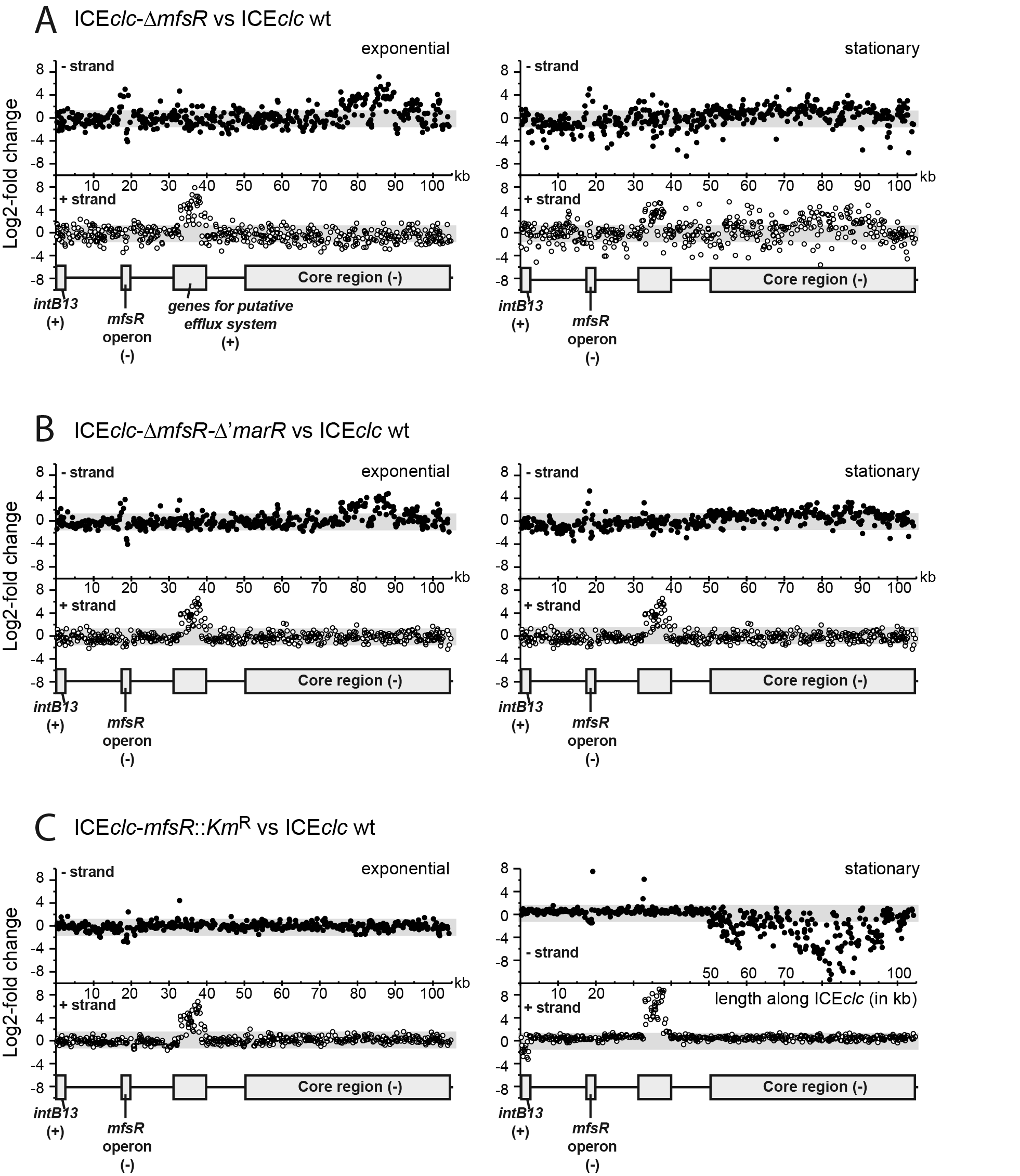

Supplement: Figure S4 — Pair-wise comparisons of expression in the ICEclc area of mfsR mutants by microarray analysis. (A) P. putida UWC1 (ICEclc-ΔmfsR, strain 4322) compared to P. putida UWC1 (ICEclc, strain 2737). (B) P. putida UWC1 (ICEclc-ΔmfsR-Δ'marR, strain 3453) versus P. putida UWC1 (ICEclc, strain 2737). (C) P. putida UWC1 (ICEclc-mfsR::KmR, strain 2961) versus P. putida UWC1 (ICEclc, strain 2737). Panels indicate comparisons of exponentially growing or stationary phase cells, with hybridization signals on the plus- (open symbols) or minus-strand (closed symbols) of ICEclc. Dots indicate the 2log-fold change of hybridization signal per microarray probe in the comparison, plotted at their distance along the ICEclc sequence (X-axis; in kb). A scheme of ICEclc is redrawn at the bottom of each section, with regions of interest as grey boxes (+ or - indicate the DNA strand on which the region is encoded). Grey bars in the background indicate the two-fold cut-off level. (TIF) [file pgen.1004441.s004.tif]

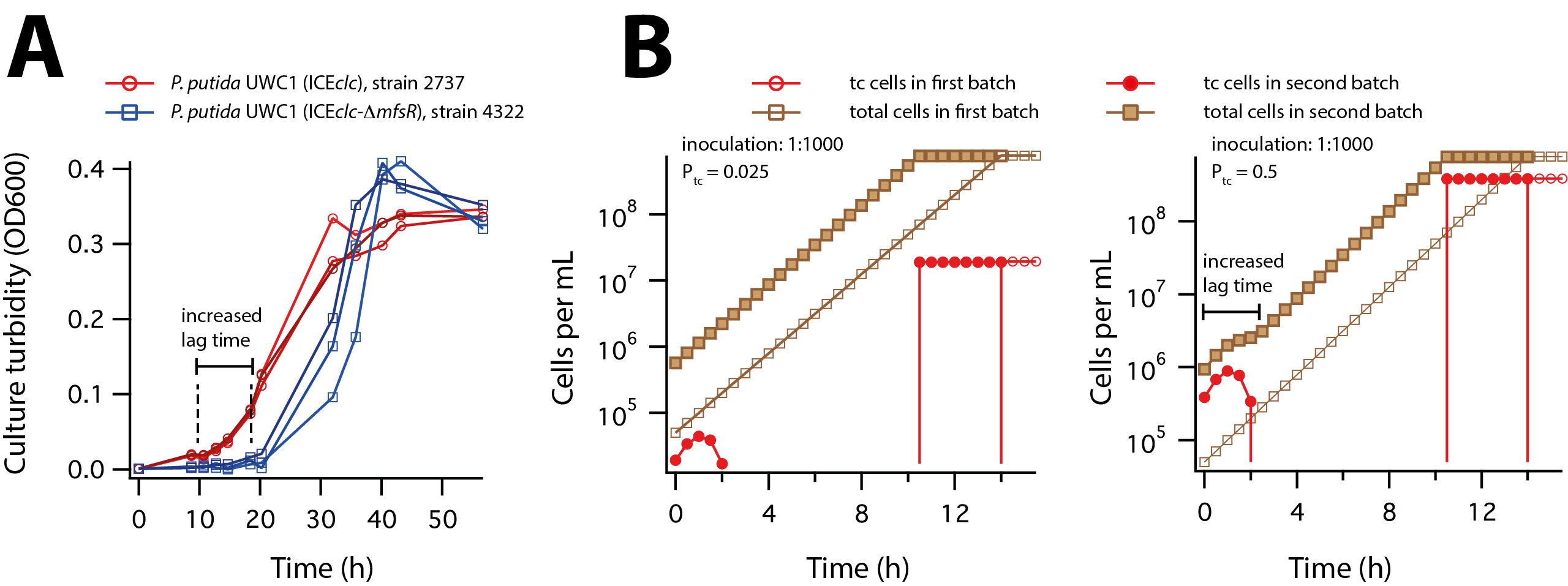

Supplement: Figure S5 — Observed and expected population growth in cultures of P. putida with wild-type ICEclc and the ΔmfsR mutant. (A) Measured turbidities in triplicate batch cultures growing on 3CBA as sole carbon and energy source. Note the increased lag time of the P. putida ΔmfsR mutant. (B) Modeled population growth of all cells (brown) and tc cells (red) in two subsequent batch cultures growing on 3CBA; the second being inoculated with 1∶1000 volume from the first culture in stationary phase. Scenarios show predicted behavior for wild-type ICEclc (with a probability P tc of 0.025 of tc cell appearance in stationary phase) and for ΔmfsR (with P tc = 0.5). Note how a tc population appears in stationary phase, which is transferred to a new culture, but rapidly dies as a result of activation of the par-shi system [30]. Note further how exponential growth rates remain the same for both wild-type ICEclc and the ΔmfsR mutant. Modeling based on Monod kinetics, using the following parameters: total C = 4.25 mg C/ml, dry weight of a single cell = 2·10-12 g, yield = 0.3 g/g, Ks = 0.02 mg/mL, generation time of non-tc cells = 1 h, generation time of tc-cells = 2 h, death rate of tc cells = 0.4 h-1. (TIF) [file pgen.1004441.s005.tif]

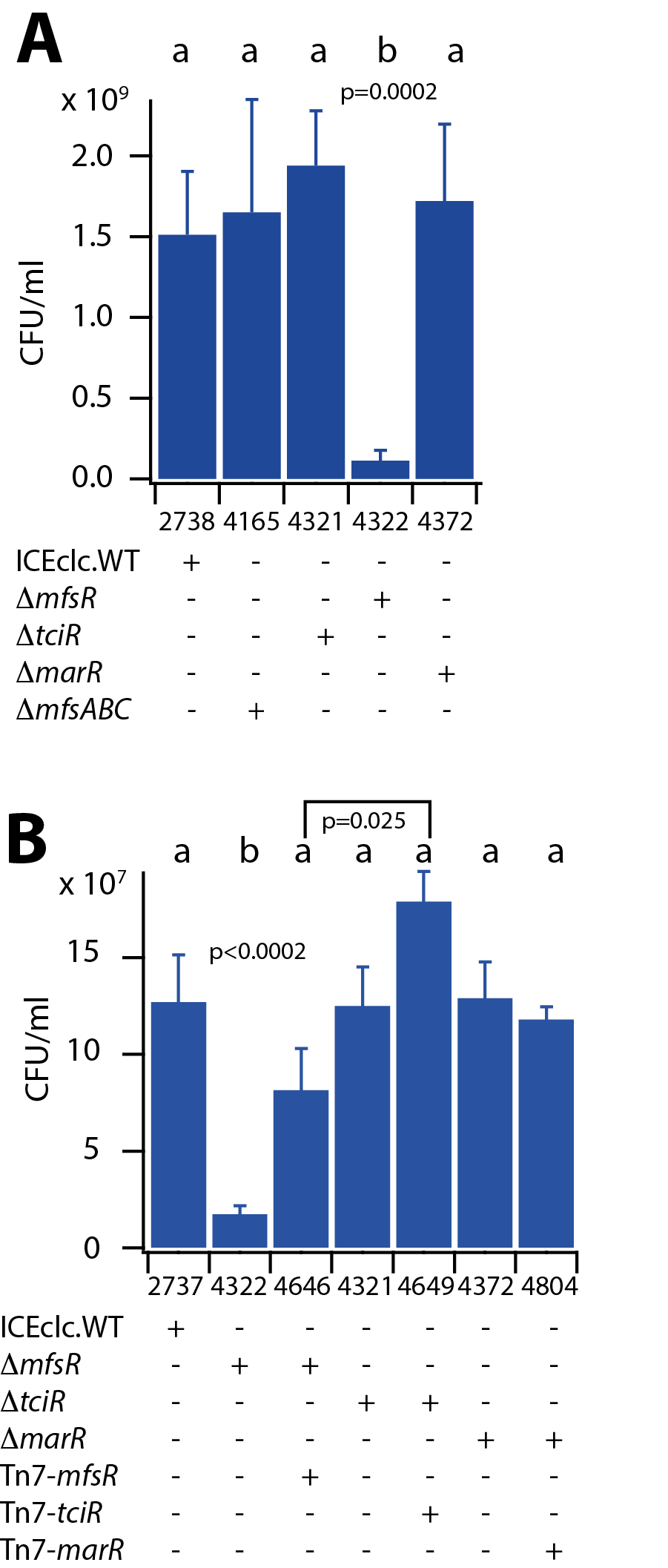

Supplement: Figure S6 — Population sizes of P. putida UWC1 carrying wild-type (2737, 2738) or mutant ICEclc, measured as colony forming units (CFU) on 5 mM 3-chlorobenzoate agar plates, per ml of resuspended culture spotted and incubated for 48 h on 0.5 mM 3-chlorobenzoate containing agar medium. (A) P. putida UWC1 strains: 4165, ICEclc with a deletion in the genes for the suspected efflux system (as unrelated control); 4321, tciR deletion; 4322, mfsR deletion; 4372, marR deletion; versus P. putida UWC1 (ICEclc), 2738. (B) P. putida UWC1 strains: 2737, ICEclc wild-type; 4321, tciR deletion; 4322, mfsR deletion; 4372, marR deletion; 4646, mfsR deletion but complemented in trans by a single copy mini-Tn7 inserted mfsR gene under its own promoter; 4649, tciR deletion but complemented in trans by a single copy mini-Tn7 inserted fragment with the (mfsRfs)-(marRfs)-tciR genes under the mfsR promoter. 4804, marR deletion but complemented in trans by a single copy mini-Tn7 inserted fragment with the (mfsRfs)-marR-(tciRfs) genes under the mfsR promoter. (A) and (B), Independently carried out experiments on different occasions and by different scientists. Letters indicate statistically indistinguishable groups identified from ANOVA tests on biological replicates, followed by Tukey's post hoc testing. P-values indicate the significance for the overall group difference (a versus b), or in one specific case, between the samples connected by the line. (TIF) [file pgen.1004441.s006.tif]
