# Supplementary material for: An Operon of Three Transcriptional Regulators Controls Horizontal Gene Transfer of the Integrative and Conjugative Element ICEclc in Pseudomonas knackmussii B13
Source: PLoS Genet. 2014 Jun 19;10(6):e1004441. doi: 10.1371/journal.pgen.1004441 (PMC4063739; doi:10.1371/journal.pgen.1004441)
Supplement: Table S1 — Oligonucleotides used for amplification of ICEclc fragments. (DOCX) [file pgen.1004441.s007.docx]

**Table S1.** Oligonucleotides used for amplification of ICE*clc* fragments.

| Primer number | Sequence 5'-3' | Purpose | Position on ICE*clc* |
| --- | --- | --- | --- |
| 070313 | CACTGTCTCCGTAGCCGA | forward primer in *clcA* gene | 13063 |
| 070315 | ATGCCAGTGCCATACCAGA | Reverse primer in *clcA* gene | 13362 |
| 101105 | gaattctgctccaggacggtgaacaa | ICE*clc*-∆*mfsR*-∆*'marR* down fragment, EcoRI | 17735-17754 |
| 101106 | ctcgagggctgctccatttggtttgact | ICE*clc*-∆*mfsR*-∆*'marR* down fragment, XhoI | 18373-18394 |
| 101107 | ctcgagGACTTGGTTAGTGCGTTGCATCT | ICE*clc*-∆*mfsR*-∆*'marR* up fragment, XhoI | 19167-19189 |
| 101108 | ggatccgcagtgcgagagttccttttagag | ICE*clc*-∆*mfsR*-∆*'marR* up fragment, BamHI | 19714-19737 |
| 101109 | ttttgaattcagcccatatgataagcaagagtga | ICE*clc*-∆*tciR* down fragment, EcoRI | 16644-16667 |
| 101110 | ttttctcgagACGACCTATCTGCTCCGAC | ICE*clc*-∆*tciR* down fragment, XhoI | 17145-17163 |
| 101111 | ttttctcgagtcacggccgtggttctgtga | ICE*clc*-∆*tciR* up fragment, XhoI | 17986-18005 |
| 101112 | ttttggatccGTGGTGACATTCATGCGTGCCTAT | ICE*clc*-∆*tciR* up fragment, BamHI | 18549-18572 |
| 120816 | ttttgaattcgccgagttcatggagcg | ICE*clc*-∆*'marR* down fragment, EcoRI | 17404-17420 |
| 120817 | ttttctcgagtgacctcgatagcaaac | ICE*clc*-∆*'marR* down fragment, XhoI | 18015-18031 |
| 120818 | ttttctcgagcgcatcaaattgctgtg | ICE*clc*-∆*'marR* up fragment, XhoI | 18469-18485 |
| 120819 | ttttggatcccaactaccgacatgatccagcgcg | ICE*clc*-∆*'marR* up fragment, BamHI | 19043-19066 |
| 120820 | ttttggatccgcagtgcgagagttccttttagag | ICE*clc*-∆*'mfsR* up fragment, BamHI | 19714-19737 |
| 120821 | ttttctcgagctgctcggtggcaaggt | ICE*clc*-∆*'mfsR* up fragment, XhoI | 19144-19160 |
| 120822 | ttttgaattctcacggccgtggttctgtga | ICE*clc*-∆*'mfsR* down fragment, EcoRI | 17986-18005 |
| 120823 | ttttctcgagacgcggcggtggtgacatt | ICE*clc*-∆*'mfsR* down fragment, XhoI | 18562-18580 |
| 070934 | aacaagccagggatgtaacg (tpnRL17-1) | Map Km^R^ insertion flanking region in transposon mutants. | not on ICE*clc* |
| 070935 | cagcaacaccttcttcacga (tpnRL13-2) | Map Km^R^ insertion flanking region in transposon mutants. | not on ICE*clc* |
| 090404 | GTCGGAGCAGATAGGTCGT | RT primer for analysis of tciR-marR-mfsR transcript | 17141 |
| 090411 | CCTGGTCGCCGTCGTGAA | RT-PCR primer in *tciR* (forward) | 17222 |
| 090412 | AGTTGCCGCGCTGAAGAG | RT-PCR primer in *tciR* (reverse) | 17626 |
| 090413 | TTCAGCGAGGGCAACAAAGTAGC | RT-PCR primer for *marR* (forward) | 17918 |
| 090414 | ACGCGGCGGTGGTGACATT | RT-PCR primer for *marR* (reverse) | 18575 |
| 090415 | CCATAGGCACGCATGAATGTCACCAC | RT-PCR primer for *mfsR* (forward) | 18542 |
| 090416 | GCAGATGCAACGCACTAACCAAGTC | RT-PCR primer for *mfsR* (reverse) | 19186 |

a) small caps, auxiliary sequence absent from ICE*clc*.

b) capital letters, ICE*clc* sequences.
